# ASC filament formation serves as a signal amplification mechanism for inflammasomes

**DOI:** 10.1038/ncomms11929

**Published:** 2016-06-22

**Authors:** Mathias S. Dick, Lorenzo Sborgi, Sebastian Rühl, Sebastian Hiller, Petr Broz

**Affiliations:** 1 Focal Area Infection Biology, Biozentrum, University of Basel, Klingelbergstrasse 50/70, 4056 Basel, Switzerland; 2 Focal Area Structural Biology, Biozentrum, University of Basel, Klingelbergstrasse 50/70, 4056 Basel, Switzerland

## Abstract

A hallmark of inflammasome activation is the ASC speck, a micrometre-sized structure formed by the inflammasome adaptor protein ASC (apoptosis-associated speck-like protein containing a CARD), which consists of a pyrin domain (PYD) and a caspase recruitment domain (CARD). Here we show that assembly of the ASC speck involves oligomerization of ASC^PYD^ into filaments and cross-linking of these filaments by ASC^CARD^. ASC mutants with a non-functional CARD only assemble filaments but not specks, and moreover disrupt endogenous specks in primary macrophages. Systematic site-directed mutagenesis of ASC^PYD^ is used to identify oligomerization-deficient ASC mutants and demonstrate that ASC speck formation is required for efficient processing of IL-1β, but dispensable for gasdermin-D cleavage and pyroptosis induction. Our results suggest that the oligomerization of ASC creates a multitude of potential caspase-1 activation sites, thus serving as a signal amplification mechanism for inflammasome-mediated cytokine production.

## Abstract (web summary)

Inflammasomes regulate IL-1β family maturation and pyroptosis. Here the authors show that ASC oligomerization and the formation of ASC specks are needed for IL-1β processing, but are not required for pyroptosis, indicating distinct inflammasome regulatory pathways.

## Graphical abstract

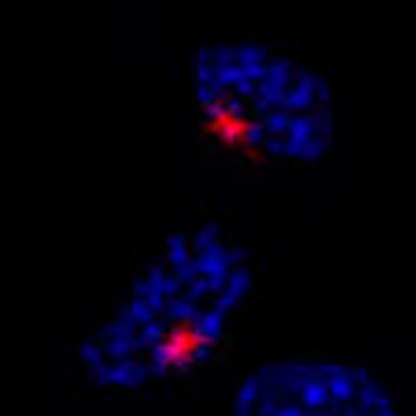

Detection of pathogens by the innate immune system relies on germline-encoded pattern recognition receptors (PRRs), which recognize a variety of pathogen-derived molecules, known as pathogen-associated molecular patterns (PAMPs), and host-derived danger signals, known as danger-associated molecular patterns^1^. Although most PRRs initiate transcriptional responses, such as the expression of cytokines^2^,^3^, a subset of cytosolic PRRs promote the assembly of inflammasome complexes and subsequent activation of the cysteine protease caspase-1 (refs 4, 5). To date, only members of the NOD-like receptor (NLR) family, the PYHIN protein family and PYRIN were shown to assemble inflammasomes in response to different cytosolic PAMPs or danger-associated molecular patterns^5^,^6^. A unifying feature of all these receptors is either a pyrin domain (PYD) or a caspase recruitment domain (CARD), both of which belong to the death-fold domain superfamily. On the basis of the presence of these domains, the receptors can be classified as PYD-containing receptors (NLRP3: NLR family, PYD containing 3; AIM2: absent in myeloma 2; pyrin) or CARD-containing receptors (NLRC4: NLR family, CARD containing 4; NLRP1: NLR family, PYD containing 1). After receptor activation and oligomerization, these domains recruit the adaptor protein ASC (apoptosis-associated speck-like protein containing a CARD) and pro-caspase-1 into the complex through homotypic domain–domain interactions. Within the inflammasome, pro-caspase-1 is activated by dimerization and auto-proteolysis, and the proteolytically active hetero-tetramer is released^7^. Several substrates of caspase-1 have been identified, among them the pro-forms of the cytokines interleukin (IL)-1β and IL-18 (refs 7, 8, 9). Another consequence of inflammasome activation is the induction of a pro-inflammatory cell death called pyroptosis^10^. Pyroptosis is driven by the amino-terminal cleavage fragment of gasdermin-D, a protein cleaved by caspase-1, and results in the lysis of the host cell and subsequent release of cytoplasmic content, among them processed IL-1β and IL-18 (refs 11, 12).

Recent studies have started to elucidate the role of the inflammasome adaptor protein ASC^13^,^14^,^15^, a bipartite protein composed of a PYD and a CARD, also known as PYCARD^16^,^17^. ASC has previously been regarded as a simple adaptor protein that links PYD-containing receptors to the CARD-containing caspase-1, via homotypic PYD–PYD and CARD–CARD interactions^18^. However, its role appears to be more complex, as on receptor activation ASC also forms the so-called ASC speck, a macromolecular form of the inflammasome with a diameter of ∼1–2 μm^19^,^20^. Formation of the ASC speck is independent of caspase-1 activity, but requires the oligomerization of ASC into large insoluble aggregates^20^,^21^. These ASC aggregates are stable and have even been shown to be released into the extracellular space, after pyroptosis induction, where they can trigger prolonged inflammasome activation in phagocytic cells^22^,^23^. Analyses of stimulated emission depletion microscopy images and electron micrographs suggest an irregular, filamentous shape^22^,^23^. Consistently, we and others have shown that ASC oligomerizes into long helical filaments via its PYD^13^,^14^,^15^. Despite the advances regarding the atomic structure of the ASC filament, whether the ASC speck is the functional unit of the inflammasome is unclear. Several inflammasome receptors (for example, NLRC4 and mouse Nlrp1b) contain a CARD instead of a PYD and are able to recruit caspase-1 even in the absence of ASC. Indeed, we and others have shown that ASC is not required for cell death induction by these receptors^24^,^25^,^26^,^27^,^28^. Nonetheless, activation of CARD-containing receptors results in ASC speck formation in wild-type (WT) cells, but how their CARD initiates ASC filaments is unknown. Furthermore, it is unclear whether ASC oligomerization itself is required for inflammasome signalling. Previous mutagenesis studies of ASC could not address the role of ASC oligomerization in signalling, as they were based on overexpression of ASC mutants in HEK293 cells, which is prone to artefact generation and lacks the means to study the effects on downstream signalling^13^,^29^,^30^.

Here we use retroviral expression of ASC mutants in *Asc*-deficient immortalized mouse macrophages, to address the mechanism of ASC speck formation and the functional relevance of ASC filament formation for inflammasome signalling. Our results reveal an architectural role for the ASC^CARD^, showing that it is required to link individual ASC filaments towards forming the dense ASC speck. Furthermore, we show that ASC bridging molecules are necessary to allow CARD-containing receptors the initiation of ASC filament formation. Finally, we use site-directed mutagenesis of the ASC^PYD^–ASC^PYD^ interaction interfaces to identify mutations that disrupt ASC filament formation without affecting the interaction to receptor PYDs. Surprisingly, such ASC mutants are still able to initiate caspase-1-dependent gasdermin-D maturation and subsequent cell death, but lack the ability to form filaments, to assemble ASC specks and to process IL-1β, thus uncoupling the two major downstream signalling pathways. In conclusion, our data show that the ASC speck has a function in inflammasome signalling and support a model in which the rapid oligomerization of ASC via its PYD creates a multitude of potential caspase-1 activation sites, thus serving as a signal amplification mechanism for inflammasome signalling.

## Results

### Distinct roles for the ASC^PYD^ and ASC^CARD^ in speck assembly

**Figure 1:**
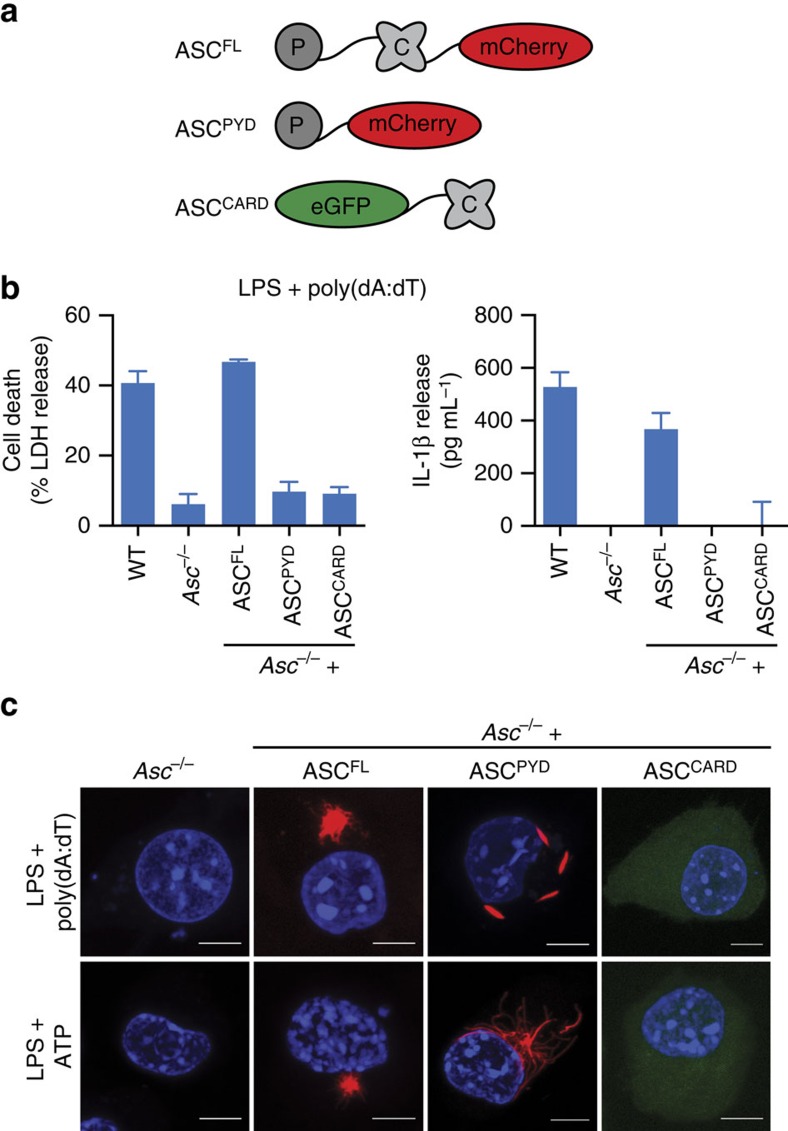
Both domains of ASC are required for signalling. (**a**) Schematic representation of the domain organization of fluorophore-tagged (mCherry or enhanced GFP (eGFP)) WT ASC (ASC^FL^), ASC^PYD^ and ASC^CARD^ constructs. (**b**) Release of LDH (assessing cell death) and IL-1β from LPS-primed immortalized WT, *Asc*^*−/−*^ or *Asc*^*−/−*^ BMDMs expressing ASC^FL^, ASC^PYD^ or ASC^CARD^ 3 h after poly(dA:dT) transfection (1 μg ml^−1^). (**c**) Representative images of cell lines from (**b**) 3 h after poly(dA:dT) transfection (1 μg ml^−1^) or 1 h after ATP treatment (5 mM). DNA (blue, Hoechst), ASC^FL^ or ASC^PYD^ (red) and ASC^CARD^ (green). Scale bars, 10 μm. Data (**b**,**c**) are representative of three independent experiments. Graphs show the mean and s.d. from quadruplicate wells. See also Supplementary Fig. 1.

Although ASC is the main structural component of the ASC speck^20^,^24^, conflicting reports have implicated either the ASC^PYD^ or the ASC^CARD^ in speck assembly^13^,^31^,^32^. To address the role of the ASC^PYD^ and the ASC^CARD^ in speck formation and downstream signalling, we transduced immortalized *Asc*^*−/−*^ murine bone marrow-derived macrophages (BMDMs) with fluorescently tagged full-length ASC (ASC^FL^) or ASC^PYD^ and ASC^CARD^ alone (Fig. 1a). As overexpression of ASC^FL^ can result in aggregation even in the absence of activated receptor^19^, we selected individual clonal lines that did not display any autoactivation when unstimulated (Supplementary Fig. 1a). To test the functionality of these constructs, cells were primed with lipopolysaccharide (LPS) and transfected with the synthetic DNA analogue poly(deoxyadenylic-deoxythymidylic) acid (poly(dA:dT)), an activator of AIM2 (refs 33, 34). mCherry-tagged ASC^FL^, expressed in *Asc*^*−/−*^ cells, promoted pyroptosis (as measured by the release of lactate dehydrogenase (LDH)) and IL-1β release (measured by enzyme-linked immunosorbent assay (ELISA)), to levels seen in immortalized WT macrophage controls (Fig. 1b). We also observed comparable levels of IL-1β/-18 and caspase-1 processing and release by western blotting (Supplementary Fig. 1b). In contrast, neither the ASC^PYD^ nor ASC^CARD^ restored cell death, IL-1β/-18 secretion or caspase-1 cleavage on AIM2 activation (Fig. 1b and Supplementary Fig. 1b). Similarly, expression of the individual domains did not restore inflammasome signalling in response to extracellular ATP, an NLRP3 activator^35^ (Supplementary Fig. 1c,d).

Heterologous expression of ASC^PYD^ and ASC^CARD^ or CARDs of mitochondrial antiviral-signalling protein (MAVS), retinoic acid-inducible gene 1 (RIG-I) and pro-caspase-1 results in the formation of filaments of varying length in different cell types and *in vitro*^13^,^36^,^37^,^38^,^39^,^40^. However, it is possible that such ASC^PYD^ or ASC^CARD^ filaments are overexpression artefacts, as ASC^FL^ normally forms a distinct dense speck structure^20^. To assess the ability of ASC^PYD^ and ASC^CARD^ to form macromolecular assemblies, we examined the above described cell lines by microscopy, following inflammasome stimulation. As expected, activation of AIM2 or NLRP3 induced the formation of perinuclear specks in cells expressing mCherry-tagged ASC^FL^ (Fig. 1c and Supplementary Fig. 1e,f). In line with the reported ability of ASC to form filaments^13^,^15^, specks formed by ASC^FL^ had a dense core with emanating filaments (Fig. 1c). Instead of forming similarly dense specks, the ASC^PYD^ assembled into filaments on the engagement of NLRP3 or AIM2 (Fig. 1c). Of note, filaments formed after NLRP3 or AIM2 activation differed in their appearance, possibly due to distinct modes of how NLR and PYHIN family members assemble complexes and initiate ASC filaments^13^,^41^. Remarkably, these filaments had varying lengths and widths, with some appearing clearly thicker than 90 Å, the reported diameter of an ASC^PYD^ filament^13^,^15^. Unlike reported before, we observed that the ASC^CARD^ was not able to form macromolecular assemblies (Fig. 1c and Supplementary Fig. 1e,f). Taken together, these data suggest that although both domains of ASC are necessary for signalling, only the ASC^PYD^ forms filaments on inflammasome activation. This is consistent with reports by us and others showing that full-length ASC forms helical filaments *in vitro* via the ASC^PYD^ (refs 13, 15), whereas the ASC^CARD^ is exposed on the surface and not involved in ASC filament formation^15^. As straight filaments are formed by ASC^PYD^ and compact specks are formed by ASC^FL^, our data also suggest that the ASC^CARD^ must contribute significantly to the macroscopic structure of the ASC speck.

### ASC^CARDs^ condense ASC^PYD^ filaments into ASC specks

**Figure 2:**
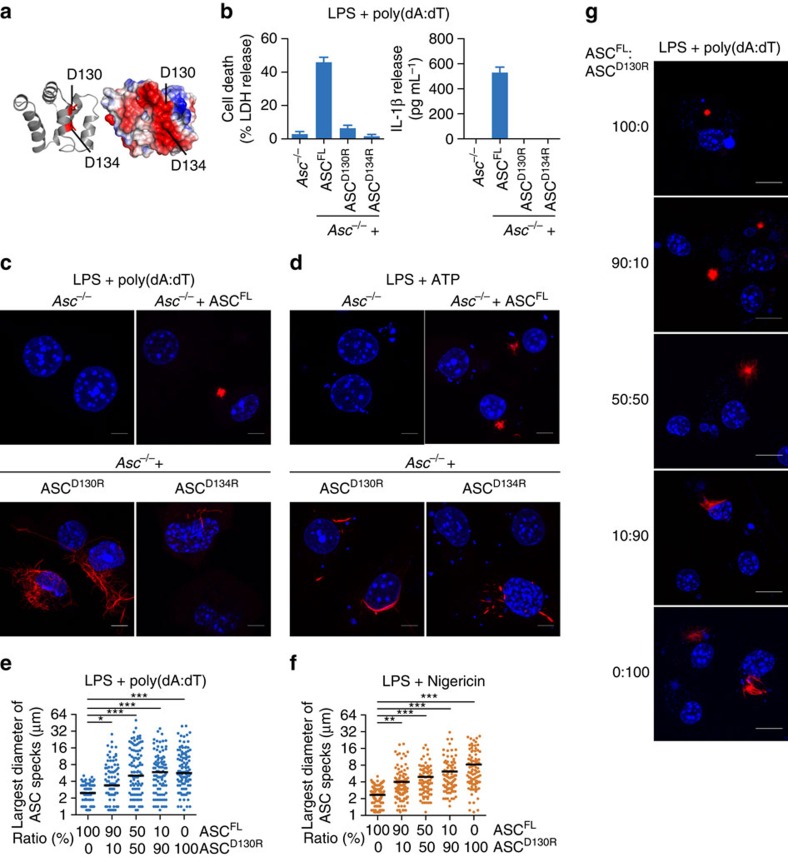
The CARD of ASC condenses PYD filaments into the speck. (**a**) Structural model of the mouse ASC^CARD^ based on the human homologue (PDB 2KN6 (ref. 67)). The structure is shown in ribbon (left) and electrostatic surface representation (right, blue, positive charge; red, negative charge). Residues D130 and D134, involved in the interaction with pro-caspase-1, are highlighted^31^. (**b**) Release of LDH and IL-1β from LPS-primed immortalized *Asc*^*−/−*^ BMDMs and *Asc*^*−/−*^BMDMs expressing ASC^FL^, ASC^D130R^ or ASC^D134R^ 3 h after poly(dA:dT) transfection (1 μg ml^−1^). (**c**,**d**) Representative images of cell lines from (**b**) 3 h after poly(dA:dT) transfection (1 μg ml^−1^) (**c**) or 1 h after ATP treatment (5 mM) (**d**). DNA was stained with Hoechst (blue) and ASC (red). Scale bars, 10 μm. (**e**,**f**) Measurement of the ASC speck diameter in primary C57BL/6 BMDMs transduced with the indicated ratio of mCherry-tagged ASC^FL^ or ASC^D130R^ and transfected with poly(dA:dT) (3 h at 1 μg ml^−1^ (**e**)) or treated with nigericin (1 h, 20 μM (**f**)) after LPS priming. (**g**) Representative images from **e**. DNA was stained with Hoechst (blue) and ASC (red). Scale bars, 5 μm. Data are representative of three (**b**–**d**) independent experiments. Graphs show the mean and s.d. from quadruplicate wells (**b**) or triplicate coverslips (**e**,**f**). The numbers of specks measured were 99, 92, 104, 94 and 108 in (**e**) and 149, 134, 85, 98 and 95 in (**f**). *P<0.05, **P<0.01 and ***P<0.001 (one-way analysis of variance). See also Supplementary Figs 2 and 3.

To test whether ASC^CARD^ is required to form ASC specks, we generated immortalized *Asc*^*−/−*^ macrophage lines expressing ASC^D130R^ or ASC^D134R^ (Fig. 2a and Supplementary Fig. 2a), two mutations in surface-exposed residues of the ASC^CARD^ that abrogate interaction of the ASC^CARD^ with the pro-caspase-1^CARD^ (ref. 31). As expected, *Asc*^*−/−*^ macrophages expressing ASC^D130R^ or ASC^D134R^ did not activate caspase-1, release mature IL-1β or undergo pyroptosis in response to DNA transfection or ATP treatment (Fig. 2b and Supplementary Fig. 2b–d). ASC^D130R^ and ASC^D134R^ formed long filaments rather than dense ASC specks in response to AIM2 and NLRP3 activation (Fig. 2c,d and Supplementary Fig. 2e,f), similar to the ASC^PYD^ alone, thus supporting our hypothesis that a functional ASC^CARD^ is required to assemble ASC^PYD^ filaments into a speck. To confirm that these mutations not only disrupt ASC^CARD^–pro-caspase-1^CARD^ interactions but also ASC^CARD^–ASC^CARD^ interactions, we expressed enhanced green fluorescent protein (eGFP)-tagged ASC^CARD^ in the presence of reconstituted inflammasomes, formed by co-transfection of AIM2 with mCherry-tagged ASC^FL^, ASC^PYD^, ASC^D130R^ or ASC^D134R^ in HEK293T cells. Co-localization and co-immunoprecipitation assays demonstrated that GFP-ASC^CARD^ was only able to interact with WT ASC and not with ASC^D130R^ or ASC^D134R^, confirming that the two mutations blocked ASC^CARD^–ASC^CARD^ interaction (Supplementary Fig. 2g,h).

If the ASC^CARD^ organizes ASC filaments into dense specks, we speculated that increasing levels of ASC^CARD^ mutants would disrupt specks formed by WT ASC, resulting in larger but less dense ASC specks. Therefore, we retrovirally transduced various ratios of mCherry-tagged WT and mCherry-tagged ASC^D130R^ into primary WT C57BL/6 macrophages (containing endogenous ASC) and measured median speck size on the activation of AIM2 by poly(dA:dT) transfection or NLRP3 by Nigericin, a pore-forming toxin^35^. Indeed, increasing levels of ASC^D130R^ resulted in a significant increase in ASC speck size (Fig. 2e,f). As expected, microscopy analysis of these large ASC specks showed that they were also less dense and had a filamentous nature (Fig. 2g). Taken together, these results support a model in which the ASC speck observed in WT cells is composed of individual ASC filaments that are formed via their PYDs and cross-linked to each other via their CARDs (Supplementary Fig. 3a).

### NLRC4 oligomerizes ASC via a bridging ASC molecule

**Figure 3:**
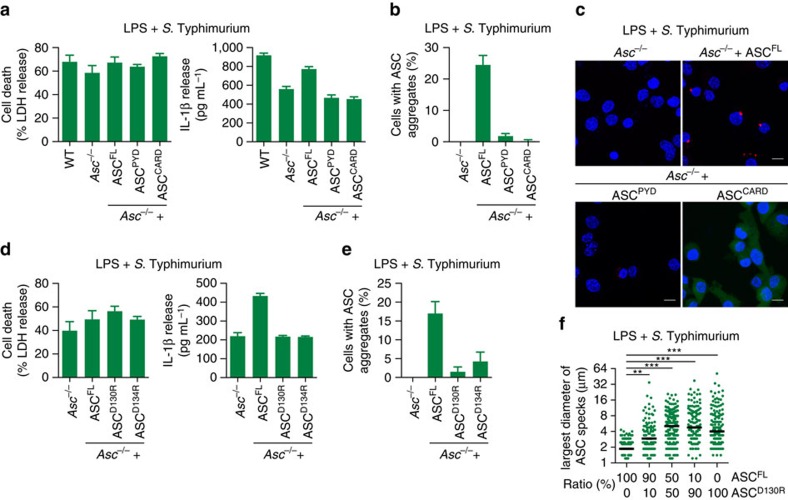
A bridging ASC molecule is required for ASC speck formation after NLRC4 activation. (**a**) Release of LDH and IL-1β from LPS-primed immortalized WT, *Asc*^*−/−*^ or *Asc*^*−/−*^ BMDMs expressing ASC^FL^, ASC^PYD^ or ASC^CARD^ after infection with log-phase WT *S.* Typhimurium SL1344 (multiplicity of infection (MOI) 10 for 1 h). (**b**) Quantification of the percentage of cells from **a** with ASC specks or filaments (collectively referred to as ASC aggregates). (**c**) Representative images of cell lines from **b**. DNA (blue, Hoechst) and ASC (red). Scale bars, 10 μm. (**d**) Release of LDH and IL-1β from LPS-primed *Asc*^*−/−*^ BMDMs and *Asc*^*−/−*^ BMDMs expressing ASC^FL^, ASC^D130R^ or ASC^D134R^ after infection with log-phase WT *S.* Typhimurium SL1344 (MOI 10 for 1 h). (**e**) Quantification of the percentage of cells with ASC aggregates from **d**. (**f**) Measurement of the ASC speck diameter in primary C57BL/6 BMDMs transduced with the indicated ratio of mCherry-tagged ASC^FL^ or ASC^D130R^ and infected with log-phase WT *S.* Typhimurium SL1344 (MOI 10 for 1 h) after LPS priming. Data are representative of three (**a**–**e**) independent experiments. Graphs show the mean and s.d. from quadruplicate wells in (**a**,**c**–**e**) and triplicate coverslips in **f**. The numbers of specks measured were 194, 134, 184, 128 and 141 in **f**. *P<0.05, **P<0.01 and ***P<0.001 (one-way analysis of variance). See also Supplementary Fig. 3.

Most inflammasome-forming receptors contain a PYD and initiate ASC filaments directly by homotypic PYD–PYD interactions. Notable exceptions are NLRC4 and mouse Nlrp1b, which contain a CARD and thus might recruit and activate caspase-1 directly^42^,^43^. Nevertheless, ASC promotes caspase-1 processing and efficient IL-1β/-18 release after NLRC4 and Nlrp1b activation^26^,^44^. ASC specks can be observed after NLRC4 activation, but how CARD-containing receptors initiate oligomerization of ASC is unknown. Theoretically, NLRC4 could initiate ASC oligomerization either by (i) NLRC4^CARD^–ASC^CARD^ interaction and ASC^CARD^ oligomerization, (ii) heterotypic interaction between related death-fold domains, that is, by a non-canonical NLRC4^CARD^–ASC^PYD^ interaction or (iii) via bridging ASC molecules that would be linked with NLRC4 via CARD–CARD interactions and provide their free PYDs as seeds for ASC^PYD^ filament formation. To determine which domain of ASC was necessary for ASC speck formation after NLRC4 activation, we infected the above described cell lines harbouring the single domains of ASC with log-phase *Salmonella enterica* serovar Typhimurium SL1344 (*Salmonella* Typhimurium), a robust activator of NLRC4 (refs 45, 46). Consistent with published reports^24^,^27^,^47^, immortalized *Asc*^*−/−*^ BMDMs still induced cell death, whereas efficient IL-1β processing and release required ASC (Fig. 3a). Expression of ASC^FL^ in *Asc*^*−/−*^ cells restored IL-1β release, but neither the ASC^PYD^ nor ASC^CARD^ alone were able to functionally restore inflammasome activation (Fig. 3a). We next analysed whether the ASC^PYD^ or ASC^CARD^ would form specks or filaments after NLRC4 activation by microscopy. Although ASC^FL^ formed specks, ASC^PYD^ alone did not initiate filaments following activation of NLRC4 (Fig. 3b,c), which was in contrast to what we had observed for PYD-containing receptors (Fig. 1c). In addition, cells expressing ASC^CARD^ alone did not form any specks or macromolecular filaments, again suggesting that the ASC^CARD^ in the endogenous setting is not able to form filaments (Fig. 3b,c). These results exclude the possibilities that ASC specks form via CARD oligomerization after NLRC4 activation, or that a heterotypic NLRC4^CARD^–ASC^PYD^ interaction initiates ASC filament formation. Instead, they suggest that one or several bridging molecules of ASC are required to initiate ASC filament formation after NLRC4 activation, and that in these conditions ASC oligomerization into filaments and specks also proceeds via the ASC^PYD^.

We also examined cells expressing ASC^D130R^ and ASC^D134R^ mutants, which are defective for CARD–CARD interactions (Supplementary Fig. 2g,h (ref. 31)). As expected, expression of these mutants in *Asc*^*−/−*^ macrophages did not complement the release of mature IL-1β after NLRC4 activation (Fig. 3d). Furthermore, we did not observe the formation of filaments or specks in cells expressing ASC^D130R^ or ASC^D134R^ (Fig. 3e), confirming the requirement for a functional ASC^CARD^ for ASC oligomerization by NLRC4. Finally, we also tested our notion that the ASC^CARD^ is required to condense ASC filaments into a speck in the context of NLRC4 activation. Primary WT C57BL/6 BMDMs (containing endogenous ASC, thus enabling initiation of ASC oligomerization) were retrovirally transduced with varying ratios of mCherry-tagged ASC^FL^ or ASC^D130R^ and the speck diameter was measured on *S.* Typhimurium infection (Fig. 3f). The increase in speck size concurring with increasing concentrations of the ASC^D130R^ mutant indicated that also in the NLRC4 inflammasome the ASC^CARD^ condenses the ASC filaments into the speck. In conclusion, our results suggested a triple functional role for ASC^CARD^ in the NLRC4 inflammasome: (1) mediating the interaction with pro-caspase-1 (refs 17, 31), (2) condensing ASC^PYD^ filaments into the speck and (3) initiating ASC oligomerization through bridging ASC molecules (Supplementary Fig. 3b).

### Pyroptosis induction is independent of ASC oligomerization

Having elucidated the general architecture of the ASC speck, we next investigated whether the ASC speck constitutes the active, signalling-competent inflammasome. Higher-order signalosomes were reported for other innate immune signalling pathways (Toll-like receptors, receptor-interacting serine/threonine-protein kinase 2 (RIP2K) and MAVS) and could promote signal amplification and digital all-or-nothing responses^48^, leading us to hypothesize that ASC oligomerization and filament formation might have a similar function for inflammasome signalling.

**Figure 4:**
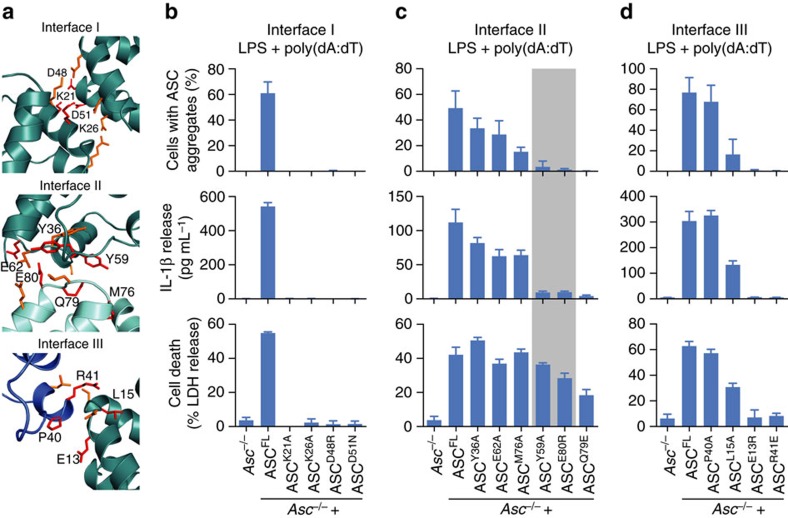
Mutations in interface II uncouple speck formation and IL-1β release from cell death. (**a**) Detailed view of the three interaction interfaces forming the ASC^PYD^ filament (PDB 2N1F (ref. 15), shown in Supplementary Fig. 4a). The polypeptide backbones are shown in ribbon representation. All amino acid side chains involved in intersubunit contacts are shown as stick models. Residues mutated in this study are coloured red with their sequence label. (**b**–**d**) Quantification of ASC aggregates or the release of LDH and IL-1β from LPS-primed immortalized *Asc*^*−/−*^ BMDMs and *Asc*^*−/−*^ BMDMs expressing ASC^FL^ or the indicated ASC mutants 3 h after poly(dA:dT) transfection (1 μg ml^−1^). ASC^Y59A^ and ASC^E80R^ are highlighted in grey. Graphs show means and s.d. from quadruplicate wells or ten random fields of view. Data are representative of at least three independent experiments. See also Supplementary Figs 4 and 6.

Within the ASC^PYD^ filaments that form the backbone of ASC specks in human and murine cells (Supplementary Fig. 4a (refs 13, 15)), individual ASC^PYDs^ interact with other six adjacent ASC^PYDs^ through three asymmetric interfaces, types I–III (Fig. 4a). Mutations targeting these interfaces can abrogate ASC filament formation *in vitro* or in cell overexpression systems^13^,^29^,^49^. As these mutations also most probably abrogate the interaction between the receptor^PYD^ and ASC^PYD^, and/or were only tested in an artificial overexpression system, a definite confirmation that ASC filament formation is required for inflammasome signalling is still missing^50^. Identification of mutations that abrogate ASC oligomerization but leave ASC–receptor interaction intact allowed us to characterize the role of ASC filaments in inflammasome signalling. Therefore, we generated a number of clonal immortalized *Asc*^*−/−*^ macrophage lines expressing different mCherry-tagged ASC mutants targeting all three interaction interfaces (Fig. 4a and Supplementary Fig. 4b–d). Next, we assessed the effects of these mutations on ASC speck/filament formation on AIM2 activation by poly(dA:dT) transfection. Mutations targeting interface I (that is, K21A, K26A, D48N and D51R) completely abrogated the filament formation as determined by microscopy (Fig. 4b upper panel and Supplementary Fig. 4e). This is consistent with interface I being the most extensive interface and required for the propagation of single layers of the filament^13^,^15^. In contrast, mutations in interfaces II and III displayed a larger variability, ranging from no defect in ASC speck formation (Y36A, E62A (interface II) or P40A (interface III)), intermediate phenotypes (M76A (interface II) or L15A (interface III)) to complete abrogation in ASC speck formation (Y59A, Q79E, E80R (interface II) or E13R and R41E (interface III)) (Fig. 4c,d upper panels and Supplementary Fig. 4e). Taken together, these data suggest that the formation of ASC specks requires all three interfaces of ASC^PYD^.

We next tested how these mutations affect downstream inflammasome signalling by measuring IL-1β secretion (Fig. 4b–d middle panels) and pyroptosis (Fig. 4b–d lower panels). In line with the loss of ASC speck formation, interface I proved to be essential for induction of downstream inflammasome signalling (Fig. 4b). Macrophages expressing mutants in interface III of ASC^PYD^ displayed similar phenotypes as interface I mutations: the ability to form ASC specks correlated with both IL-1β secretion and pyroptosis, that is, mutants either lost all inflammasome signalling (E13R and R41E), were partially affected (L15A) or not affected at all (P40A) (Fig. 4d). These results show that interface III is important for induction of downstream inflammasome signalling, albeit some mutations are tolerated.

Mutants in interface II displayed a differential signalling phenotype (Fig. 4c). Although some mutations had no or only a very small effect on inflammasome signalling (Y36A, E62A and M76A) or abrogated inflammasome signalling altogether (Q79E), we identified two mutations (Y59A and E80R) that retained the ability to induce cell death, while losing the ability to release mature IL-1β and to form ASC specks in response to AIM2 stimulation (Fig. 4c middle and lower panels, highlighted in grey). Of note, the ELISA assay displays a higher sensitivity for the mature form of IL-1β than the pro-form, thus pro-IL-1β, which is potentially released into the supernatant by pyroptosis, is poorly detected (Supplementary Fig. 5). To test whether the phenotype of the different mutants is specific to the AIM2 inflammasome, we analysed ASC speck formation, secretion of mature IL-1β and pyroptosis in response to NLRP3 stimulation by ATP treatment. *Asc*^*−/−*^ macrophages expressing the ASC interface I, II and III mutants showed the same phenotype for NLRP3 stimulation as for AIM2 stimulation (Supplementary Fig. 6a–c). Importantly, also ASC^Y59A^ and ASC^E80R^ mutations abrogated speck formation and IL-1β secretion but still promoted pyroptosis on NLRP3 stimulation (Supplementary Fig. 6b, highlighted in grey). Finally, we also examined IL-1β release and pyroptosis of macrophages expressing interface II mutants treated with *Clostridium difficile* Toxin B (TcdB). TcdB was recently described to activate the pyrin inflammasome, which senses modifications of Rho GTPases^6^. Again, *Asc*^*−/−*^ macrophages expressing ASC^Y59A^ or ASC^E80R^ were competent for cell death induction but could not efficiently release IL-1β (Supplementary Fig. 6d, highlighted in grey). In conclusion, our site-directed mutagenesis approach identified two mutations, Y59A and E80R, in the inflammasome adaptor ASC that uncouple pyroptosis from ASC speck formation and cytokine secretion.

### ASC oligomerization mutants maintain receptor interaction

**Figure 5:**
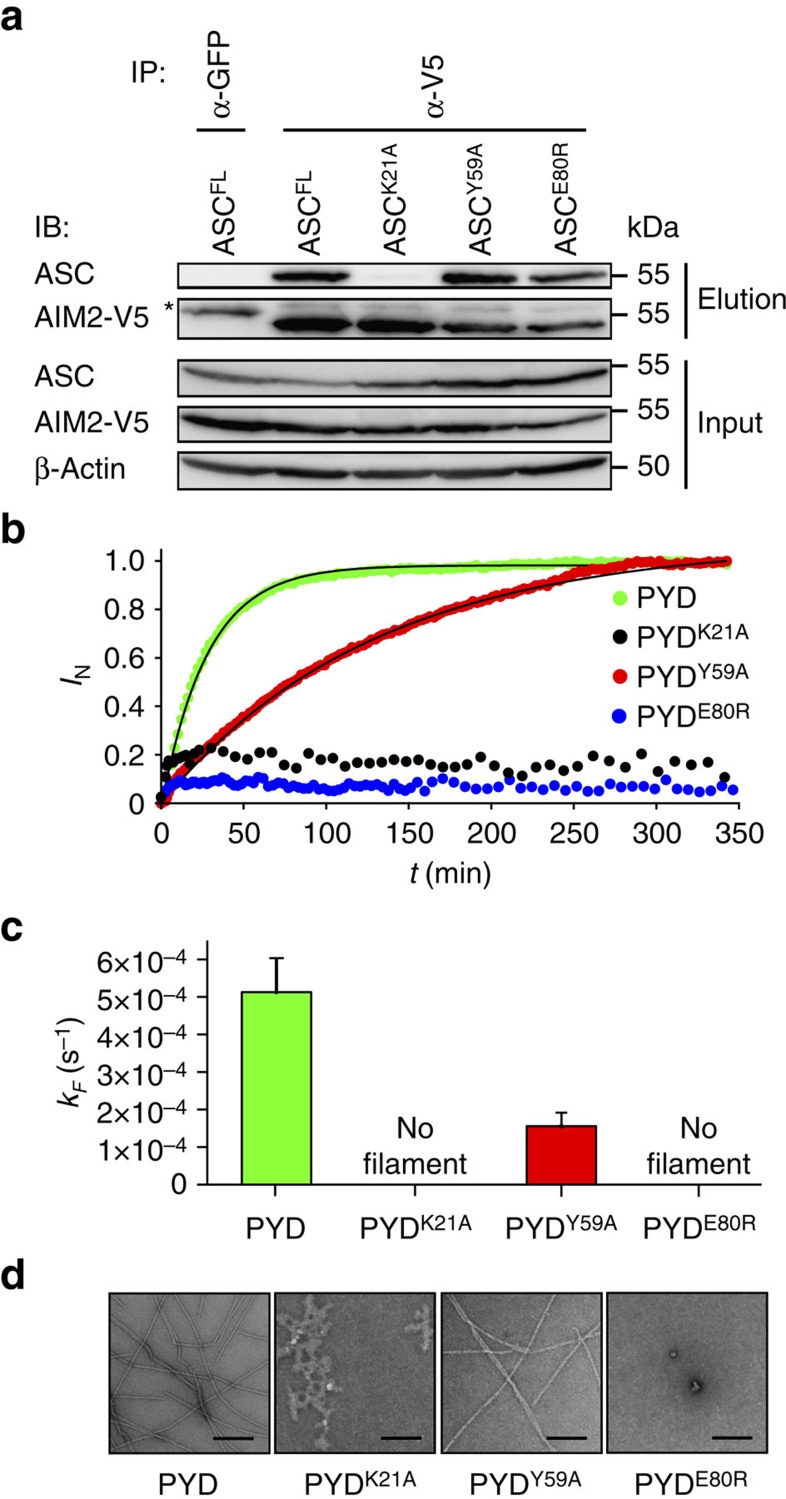
ASC–receptor interaction and ASC filament formation can be uncoupled genetically. (**a**) Western blot analysis of the interaction of AIM2 with ASC^FL^ or the indicated ASC mutants. AIM2-V5 was immunoprecipitated from lysates of HEK293T cells co-transfected with AIM2-V5 and the indicated ASC mutants. Co-immunoprecipitating proteins were identified using anti-ASC and anti-V5. *Immunoglobulin heavy chain. Results shown are representative from two independent experiments. (**b**) Filament formation of WT ASC^PYD^ and its single amino-acid variants K21A, Y59A and E80R *in vitro* monitored by dynamic light scattering. Normalized growth signals (*I*_N_) are reported as a function of time for one representative experiment for each variant (dots). Best fits to single exponential functions are shown with solid lines. (**c**) Kinetic rate constants *k*_F_ of filament formation obtained from three independent experiments. (**d**) Representative negative-stained TEM micrographs of filament formed by ASC^PYD^ and its variants after 350 min of incubation at physiological pH condition. Scale bars, 200 nm. See also Supplementary Figs 7 and 9.

As cell death was still observed in cells expressing ASC^Y59A^ and ASC^E80R^, we speculated that the mutant proteins retained the ability to interact with the PYD of the receptor, but lost the ability to oligomerize into ASC filaments. To investigate this, we reconstituted the AIM2 inflammasome by co-expressing V5-tagged AIM2 with ASC^FL^ or the ASC^K21A^, ASC^Y59A^ or ASC^E80R^ mutants in HEK293T cells. AIM2 interactors were then immunoprecipitated using an anti-V5 antibody and analysed by western blotting (Fig. 5a). Consistent with our notion, ASC^Y59A^ and ASC^E80R^ retained the ability to interact with AIM2, similar to WT ASC. In contrast, ASC^K21A^ did not interact with AIM2, possibly explaining the clear-cut phenotypes of interface I mutants (Fig. 4b and Supplementary Fig. 6b).

Given that ASC^Y59A^ and ASC^E80R^ mutants still interacted with the receptor (Fig. 5a), but did not form any ASC filaments/specks (Fig. 4c and Supplementary Fig. 4e), we speculated that the mutations abolished or reduced the ability of ASC to organize itself into filaments following the recruitment to the activated receptor. Based on our previous observation that purified ASC^PYD^ or ASC^FL^ form filaments *in vitro*^15^, we established an assay to monitor the kinetics of this oligomerization process by dynamic light scattering and analysed the kinetics of filament formation for WT ASC^PYD^ and the above-mentioned mutants (Fig. 5b,c and Supplementary Fig. 7a,b). Although WT PYD rapidly formed well-structured filaments, the K21A mutant was no longer able to oligomerize correctly and formed unspecific aggregates (Fig. 5d). Interestingly, E80R also lost the ability to oligomerize into ordered filaments, whereas the Y59A mutant formed well-ordered filaments, but at a much slower rate than the WT protein (Fig. 5b–d and Supplementary Fig. 7a,b). Thus, these results confirmed that the ASC–receptor interaction and the ASC filament formation can be uncoupled, and that the ability to rapidly form filaments/specks strongly correlates with the levels of cytokine production but not cell death.

### Gasdermin-D cleavage is independent of ASC oligomerization

**Figure 6:**
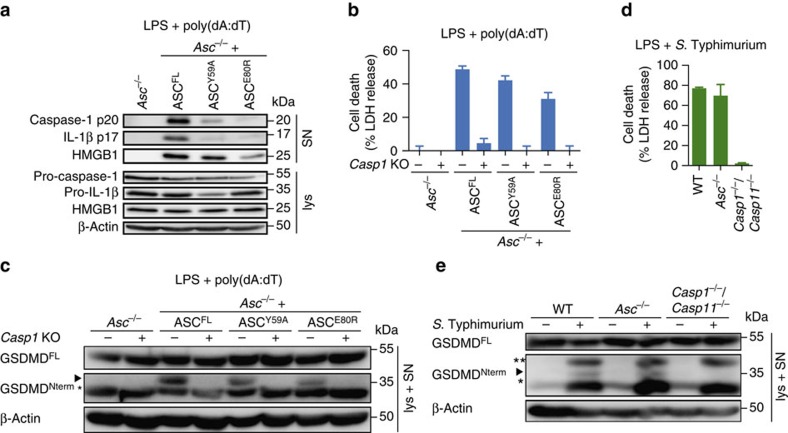
Caspase-1 but not gasdermin-D processing depends on ASC oligomerization. (**a**) Western blot analysis for cleaved caspase-1 p20, IL-1β p17, and HMGB-1 in cell supernatants (SN) and pro-caspase-1, pro-IL-1β and HMGB-1 in cell lysates (lys) of LPS-primed immortalized *Asc*^*−/−*^ BMDMs or *Asc*^*−/−*^ BMDMs expressing ASC^FL^, ASC^Y59A^ or ASC^E80R^ 3 h after poly(dA:dT) transfection (1 μg ml^−1^). (**b**) Release of LDH from LPS-primed immortalized *Asc*^*−/−*^ BMDMs or *Asc*^*−/−*^ BMDMs expressing ASC^FL^, ASC^Y59A^ or ASC^E80R^, or derived *Casp1* knockouts 3 h after poly(dA:dT) transfection (1 μg ml^−1^). (**c**) Western blot analysis for processing of full-length gasdermin-D (GSDMD^FL^) into the active N-terminal fragment (GSDMD^N-term^) in combined lysates and supernatants (lys + SN) of LPS-primed immortalized *Asc*^*−/−*^ BMDMs expressing ASC^FL^, ASC^Y59A^ or ASC^E80R^, or derived *Casp1* knockouts 3 h after poly(dA:dT) transfection (1 μg ml^−1^). Arrowhead, gasdermin-D^Nterm^ p30; *a cross-reacting band. (**d**) Release of LDH from LPS-primed primary C57BL/6 WT (WT), *Casp1*^*−/−*^*/Casp11*^*−/−*^ or *Asc*^*−/−*^ BMDMs infected with *S.* Typhimurium (multiplicity of infection (MOI)=10, 1 h). (**e**) Western blot analysis for processing of full-length gasdermin-D (GSDMD^FL^) into the active N-terminal fragment (GSDMD^N-term^) in combined lysates and supernatants (lys+SN) of LPS-primed primary C57BL/6 WT (WT), *Casp1*^*−/−*^*/Casp11*^*−/−*^ or *Asc*^*−/−*^ BMDMs infected with *S*. Typhimurium (MOI=10, 1 h) or left uninfected. Arrowhead, gasdermin-D^Nterm^ p30; *a cross-reacting band; ** a *S.* Typhimurium-specific cross-reactive band. See also Supplementary Figs 8 and 9.

The observation that even in the absence of ASC oligomerization inflammasome activation still promotes cell death but not cytokine release (Fig. 4c, Supplementary Fig. 4e) was reminiscent of NLRC4 activation in *Asc*^*−/−*^ macrophages^24^. In the absence of ASC, NLRC4 interacts with pro-caspase-1 to initiate cell death but not efficient caspase-1 autoprocessing and cytokine secretion. We therefore examined to what level ASC^Y59A^ and ASC^E80R^ were able to promote caspase-1 autoprocessing on activation of the AIM2 inflammasome. In contrast to cells expressing ASC^FL^, macrophages expressing the mutant proteins displayed significantly reduced levels of caspase-1 processing, as determined by western blotting for the released p20 subunit (Fig. 6a). Furthermore, caspase-1 autoprocessing correlated with the amount of released bioactive IL-1β (Figs 4c and 6a), but not with the induction of cell death (Fig. 4c) and the release of the danger signal high mobility group box 1 (HMGB1) into the cell supernatants (Fig. 6a), an alternative marker for cell lysis during inflammasome activation^51^. Similarly, results were obtained on activation of the NLRP3 inflammasome (Supplementary Fig. 8a). To exclude the possibility that the observed cell death is induced through caspase-1-independent pathways, we knocked out *Casp1* by CRISPR-Cas9-mediated gene targeting^52^,^53^ in the cell lines expressing ASC^FL^, ASC^Y59A^ or ASC^E80R^. Western blotting for pro-caspase-1 confirmed successful targeting (Supplementary Fig. 8b). *Casp1* deficiency significantly abrogated pyroptosis induction in cells expressing ASC^FL^, ASC^Y59A^ or ASC^E80R^ (Fig. 6b), thus confirming that the cell death observed in the parental cell lines was caspase-1 dependent. Taken together, these data confirm that caspase-1 processing is not a prerequisite for induction of cell death, expanding previous findings from CARD-containing inflammasome receptors^24^ to PYD-containing receptors.

Recent reports showed that a major substrate of caspase-1 responsible for pyroptosis is gasdermin-D, and that *Gsdmd*-deficiency abrogates pyroptosis^11^,^12^. Caspase-1 cleaves full-length gasdermin-D, whereby the active N-terminal fragment (gasdermin-D^Nterm^) is generated (Supplementary Fig. 8c,d), which induces pyroptotic cell death^11^,^12^. Consistently, CRISPR-Casp9-mediated *Gsdmd* knockout (Supplementary Fig. 8e) reduced cell death significantly in response to AIM2 and NLRP3 activation (Supplementary Fig. 8f,g). As ASC^Y59A^ and ASC^E80R^ mutants induced cell death, we speculated that gasdermin-D is still processed in cells expressing these mutant forms of ASC. Indeed, although gasdermin-D was not processed in *Asc*^*−/−*^ macrophages on stimulation of AIM2 or NLRP3, we observed gasdermin-D processing to its 30 kDa gasdermin-D^Nterm^ fragment in *Asc*^*−/−*^ macrophages expressing ASC^FL^, ASC^Y59A^ and ASC^E80R^ (Fig. 6c and Supplementary Fig. 8h). Furthermore, processing of gasdermin-D on AIM2 stimulation is dependent on caspase-1 in cells harbouring oligomerization-deficient ASC mutants, as *Casp1* deficiency abrogated gasdermin-D cleavage (Fig. 6c). These results indicated that gasdermin-D^Nterm^ causes the cell death observed in these mutations. As significant levels of cell death can be observed in *Asc*-deficient macrophages after NLRC4 activation^24^, but caspase-1 processing is reduced below detection levels, we next determined whether gasdermin-D is processed under these conditions. Indeed, we found that the induction of cell death in *S*. Typhimurium-infected WT, *Asc*^*−/−*^ and *Casp1*^*−/−*^/*Casp11*^*−/−*^ macrophages (Fig. 6d) correlated with detectable levels of processed gasdermin-D (Fig. 6e). Furthermore, CRISPR-Cas9-mediated *Gsdmd* knockout confirmed that gasdermin-D played an essential role in inducing pyroptosis during *S*. Typhimurium infections (Supplementary Fig. 8i). Taken together, these results confirm that even the smallest amounts of active caspase-1, as judged by the amount of processed caspase-1 p20 subunits, are sufficient to efficiently process gasdermin-D and trigger gasdermin-D-induced cell death. On the other hand, large amounts of processed, active caspase-1 are required to produce detectable amounts of mature, bioactive IL-1β as seen by the direct correlation of the level of caspase-1 processing with the levels of cytokine release. Importantly, the ability of ASC to form filaments and ASC specks correlates with caspase-1 activation and cytokine processing, thus supporting a model in which the rapid formation of ASC filaments acts as a signal amplification mechanism for inflammasomes, generating a multitude of caspase-1 activation sites and thus enabling the cells to rapidly mature IL-1β before the onset of pyroptosis.

## Discussion

**Figure 7:**
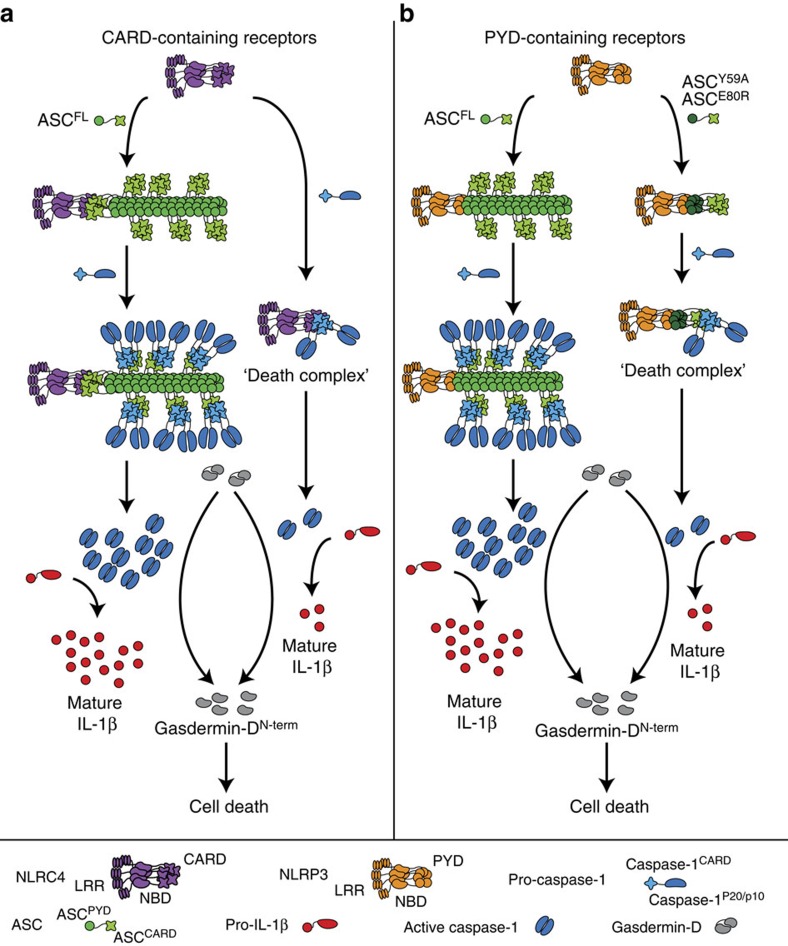
Model of signal amplification by ASC filaments. (**a**) CARD-containing receptors recruit the adaptor protein ASC via homotypic CARD–CARD interactions, that is, a bridging ASC molecule. This step nucleates the ASC^PYD^ of several bridging ASC molecules, leading to the formation of an ASC^PYD^ filament, which is condensed into the ASC speck by the ASC^CARD^. Filament formation promotes the activation of large quantities of caspase-1, thus promoting the proteolytic maturation of large amounts of cytokines (pro-IL-1β). In the absence of ASC, CARD-containing receptors directly interact with pro-caspase-1, leading to the formation of so-called ‘death complexes'. In these small complexes, only few molecules of caspase-1 are activated and pro-caspase-1 processing might not happen. The few molecules of caspase-1 are sufficient to effectively induce pyroptosis, but cytokine processing is reduced. (**b**) PYD-containing receptors can directly interact with ASC via homotypic PYD–PYD interactions, leading to ASC^PYD^ filaments and finally the ASC speck. As for CARD-containing receptors, this leads to caspase-1 activation and subsequent cytokine processing and pyroptosis. Mutations blocking or slowing ASC filament formation (for example, ASC^E80R^ or ASC^Y59A^) only allow for few molecules of caspase-1 being activated. This is sufficient to induce pyroptosis, but insufficient to produce large amounts of mature cytokines before the cell lyses.

The formation of higher-order signalling machineries, signalosomes, for transmission of receptor activation information to cellular responses is an emerging theme in signal transduction^48^. It is particularly important in innate immune signalling, where the signal generated by a few ligand–receptor interactions needs to trigger an appropriate cellular response. Formation of oligomers has been reported for different signalling adaptors, for example, B-cell lymphoma/leukemia 10 (Bcl10) or MAVS filaments, the Myddosome^36^,^54^,^55^ and others. However, the archetypical supramolecular assembly formed during inflammasome activation, the ASC speck, has remained relatively poorly understood. Here we present evidence that the formation of ASC speck by oligomerization of the inflammasome adaptor protein ASC acts as a signal amplification mechanism for inflammasomes (Fig. 7), as the rapid formation of ASC^PYD^ filaments that expose ASC^CARD^ on their surface creates a multitude of pro-caspase-1 recruitment and activation sites. We speculate that such a system might therefore be able to detect the smallest amounts of PAMPs in the host cell cytosol, as recent reports have shown that a single ligand molecule (flagellin, PrgJ) is sufficient to initiate assembly of a NLR family, apoptosis inhibitory protein (NAIP) and 10–12 NLRC4 proteins into a wheel-shaped receptor oligomer that acts as seed for ASC oligomerization^39^,^56^.

Our result reveals that this signal amplification mechanism only applies to cytokine maturation, whereas gasdermin-D-induced pyroptotic cell death could be observed even in the absence of ASC oligomerization. Why would cells need to amplify the receptor-generated signal to process IL-1β/-18? We speculate that a cell will inevitably progress towards pyroptosis, once a few molecules of caspase-1 have been activated, given the rapid kinetics by which gasdermin-D is processed^57^ and the strong toxicity of the gasdermin-D^Nterm^ fragment^11^,^12^. This fate leaves a limited time window to mature the pro-forms of IL-1β/-18 to their bioactive form. As processing of IL-1β/-18 occurs with slow kinetics, increasing the amount of active caspase-1 is a means to generate large amounts of bioactive IL-1β/-18 in the available time window. Consistent with this notion we have observed that gasdermin-D processing does not correlate with the overall level of active caspase-1. Furthermore, pyroptosis has been observed to occur within 30–60 min in stimulated cells, whereas ASC speck formation was reported to proceed much faster^23^,^58^, with all cytosolic ASC being incorporated into a speck in <3 min^20^,^59^. Thus, ASC speck formation might serve to activate sufficient amounts of caspase-1 to generate enough bioactive cytokines before the cell lyses and releases its intracellular content, including proteolytically matured cytokines.

Although recent studies revealed that ASC specks are filamentous in nature^22^,^23^, consistent with recently reported structures of human and murine ASC^PYD^ filaments^13^,^15^, it was so far unknown how these filaments assemble into a speck. Our mutagenesis studies indicates that the clustering of ASC^PYD^ filaments and finally their condensation into a dense ASC speck structure is mediated by the ASC^CARD^, which, as we have shown^15^, is exposed on the surface of the ASC^PYD^ filament. These results have uncovered a function for the ASC^CARD^ in ASC speck formation, beyond its role as adaptor domain between receptor and caspase-1. How ASC^CARDs^ condense ASC^PYD^ filaments is unknown, but it can be assumed that either dimeric interactions between ASC^CARDs^ or limited oligomerization of ASC^CARD^ from different filaments could build lattices interconnecting the ASC^PYD^ filaments. Interestingly, the same residues in ASC^CARD^ that are required for ASC–pro-caspase-1 interaction are also needed for ASC speck formation. Notably, it has previously been shown that phosphorylation of the ASC^CARD^ at Y144/Y146 is not only required for caspase-1 activation, but also for ASC speck formation^60^,^61^ Our model of the ASC speck, which includes interactions between the ASC^CARD^ domains, now provides a structural rationale for these data. To which extent ASC filaments are still formed in the absence of ASC^CARD^ phosphorylation remains to be determined.

Recently, a unified assembly mechanism was proposed for the PYD-containing receptors AIM2 and NLRP3: the receptors nucleate clusters of ASC through PYD–PYD interactions, which in turn nucleate caspase-1 filaments^13^. In analogy, it was proposed that the CARD-containing receptor NLRC4 directly nucleates caspase-1 filaments^13^,^39^, as in theory CARD-containing receptors do not require ASC for caspase-1 recruitment. Although direct recruitment of pro-caspase-1 to NLRC4 might indeed happen in *Asc*-deficient cells as we hypothesized before^24^, it does not seem to efficiently activate caspase-1, because we and others have reported that caspase-1 processing into its p20/p10 subunits and the levels of released mature cytokines are reduced. Furthermore, a large number of ASC specks can be observed in WT cells on NLRC4 activation^24^,^25^,^26^,^27^,^28^, indicating that ASC nevertheless plays an important role in NLRC4 signalling. Our results now indicate that the ASC^CARD^ lacks the ability to form higher-order oligomers at physiological concentrations, and that even on NLRC4 activation ASC speck formation relies on the ASC^PYD^. Our data thus support a model in which CARD-containing receptors initiate ASC speck formation with the help of ASC bridging molecules, and that the PYDs of these ASC molecules nucleates an ASC^PYD^ filament. Thus, the ASC^CARD^ can have a triple function in inflammasome signalling on CARD-receptor activation.

In conclusion, our study gives insights into the architecture of the ASC speck and the mechanism by which ASC filaments assemble this structure. Furthermore, we provide experimental evidence that ASC filament formation serves as an amplification mechanism in inflammasome signalling, and that this amplification serves to generate sufficient mature cytokines before the onset of pyroptotic cell death. Nevertheless, important questions remain; the higher-resolution structure of the activated NLRC4 receptor has been recently reported^39^,^56^, yet how exactly such receptor complexes with C_10_–C_12_ stoichiometry initiate the 3-start ASC^PYD^ filaments is still unknown. Furthermore, the structure of the whole ASC speck assembly, including receptor, ASC and caspase-1, is still lacking and it remains unknown whether other proteins participate in its formation. Finally, additional theoretical and experimental approaches will be necessary to understand if and how ASC speck formation might impart threshold responses, reduce biological noise and control temporal and spatial control of inflammasome signalling.

## Methods

### Cell culture

Immortalized BMDM cell lines were generated from bone marrow using infection with a v-myc/v-raf-expressing J2 retrovirus^24^,^62^. WT and *Asc*^*−/−*^ immortalized murine BMDMs were cultured in DMEM (Sigma) supplemented with 10% FCS (Bioconcept) and 10% 3T3-MCSF supernatant, and incubated at 37 °C with 5% CO_2_.

### Generation of monoclonal cell lines with mutations in ASC

Murine *Asc*^*FL*^ and *Asc*^*PYD*^ were cloned with a carboxy-terminal mCherry tag and *Asc*^*CARD*^ with an N-terminal enhanced GFP tag into V48, a derivative of the replication-defective murine stem cell retroviral construct pMSCV2.2 (excision of the IRES-GFP by EcoRI digestion, gift from Thomas Henry). Mutations were introduced by SOE PCR using appropriate oligonucleotides (Supplementary Table 1) and cloned into V48. GP2 packaging cells were transfected with the individual vectors (9 μg per 1.5 × 10^6^ cells) and the lentviral envelope vector VSV-G (Addgene, 6 μg per 1.5 × 10^6^ cells), and the retroviral particles were used to transduce *Asc*^*−/−*^ iBMDMs (10^6^ cells). Seven days later, the transduced iBMDMs were sorted into single cells based on the mCherry or enhanced GFP expression (FACS) and grown up to clonal cell lines. Up to ten clones of each cell line were tested for inflammasome activation and the level of ASC expression was assessed by western blotting and a representative clone was selected for further analysis.

### Retroviral transduction of primary BMDMs

For transduction of primary bone marrow cells, retroviral particles were generated in Phoenix-Eco packaging cells and used to transduce WT C57BL/6 bone marrow cells after 48 and 72 h of culture in medium with 10% 3T3-MCSF supernatant. Inflammasome stimulation was performed 4 days after the first transduction^24^.

### Inflammasome stimulation

Immortalized BMDMs (seeded at 250 000 cells per ml in 96-well plates) were primed for 4 h with 100 ng ml^−1^ LPS O55:B5 (Invivogen). The NLRP3 inflammasome was triggered by addition of 5 mM extracellular ATP (Sigma-Aldrich) for 60 min. The AIM2 inflammasome was triggered by transfection of 1 μg ml^−1^ of the synthetic DNA analogue poly(dA:dT) (Invivogen) using Lipofectamine 2000 (Invitrogen), according to the manufacturer's protocol, in OptiMEM (Gibco) for 3 h. The NLRC4 inflammasome was triggered by infection of the cells with *S. enterica* serovar Typhimurium SL1344 at a multiplicity of infection of 10. The infection was synchronized by centrifugation and continued for 60 min. The pyrin inflammasome was triggered by addition of 1 μg ml^−1^ (final concentration) of *C. difficile* toxin B (CdtB, Enzo Biotech) for 2.5 h.

### Cell death and IL-1β release measurements

IL-1β release was measured by ELISA (eBiosciences). Cell death was quantified by measuring LDH release using the LDH Cytotoxicity detection kit (TaKaRa Clontech). To normalize for spontaneous cell lysis, the percentage of cell death was calculated as follows: [(LDH sample)−(LDH negative control)]/[(LDH positive control)−(LDH negative control)] × 100.

### Imaging and quantification of ASC speck formation

Cells were seeded on coverslips (150 000 cells per coverslip) and treated as described above with the addition of 25 μM Z-VAD-fmk (Bachem) to prevent detachment of pyroptotic cells and therefore loss of cells with ASC specks. Coverslips were fixed with 4% paraformaldehyde (PFA) (15 min, 37 °C, Alfa Aesar) and washed with PBS. Nuclei were stained with Hoechst 33342 (Life Technologies) for 10 min and the slides mounted using Vectashield (Vector Laboratories). For quantifications of ASC aggregates (specks or filaments), ten random regions of interest were imaged at × 20 magnification (Leica DMI3000B inverted fluorescence microscope, HCX PL FLUOTAR objective, Leica DFC3000G camera and LAS AF Version 3 software) and the number of ASC aggregates and cells were counted. For representative images, the slides were imaged at × 63 magnification (Leica point scanning confocal ‘SP8', HC PL APO CS2 × 63 objective, Leica AF software version 3). For measurements of speck sizes, random regions of interest were images at × 63 magnification (Leica point scanning confocal ‘SP8') and the largest diameters of individual specks were measured using Fiji^63^.

### Western blotting

For western blotting of supernatant and lysate samples, cells were seeded at 10^6^ cells per well in six-well plates and treated as described above. Supernatants were precipitated with 10% trichloracetic acid, precipitates washed with acetone and resuspended in 40 μl 1 × SDS–PAGE sample buffer and boiled at 95 °C for 10 min. Cells were lysed with 200 μl 1 × RIPA (50 mM Tris-HCl pH 7.5, 150 mM NaCl, 0.1% SDS, 0.5% sodium deoxycholate, 1% NP-40, 1 × protease inhibitor cocktail (Roche)) for 30 min on ice. Lysates were re-suspended in 5 × SDS–PAGE sample buffer and boiled at 95 °C for 10 min^64^. For combined supernatant and lysates, samples were prepared as above, but supernatant precipitates were resuspended in lysate samples. Samples were run on 14% (supernatants or lysates) or 12% (combined SN+lysates) acrylamide gels (1 h, 170 V, 40 mA per gel), transferred to polyvinylidene difluoride membranes (1 h, 100 V constant), blocked in 5% milk in Tris-buffered saline+Tween-20 (TBS-T) and incubated with primary antibodies in 5% BSA–TBS-T for 16 h at 4 °C or 2 h at room temperature with agitation. Secondary antibodies were diluted 1:5,000 in 5% milk–TBS-T and incubated for 1 h at room temperature. The membranes were developed using either LumiGLO (KPL) or LumiGLO Reserve (KPL). The following antibodies and dilutions were used: rat anti-Caspase-1 p20 (Genentech, 1:1,000; Supplementary Figs 1b,d and 2c,d) or mouse anti-Caspase 1 p20 (1:4,000, AG20B-0042, AdipoGen; Fig. 6a and Supplementary Fig. 8a), rat anti-ASC (Genentech, 1:2,000; Fig. 5a and Supplementary Fig. 2a,h) or rabbit anti-ASC (1:1,000, AG25B-006, Adipogen, all other figures), rabbit anti-IL-18 (5180R-100, Biovision, 1:1,000), goat anti-IL-1β (AF-401-NA, R&D, 1:1,000), mouse anti-GFP (632381, Clontech, 1:1,000), mouse anti-mCherry (ab125096, Abcam, 1:1,000), mouse anti-V5 (R960-25, Invitrogen, 1:1,000), rabbit anti-HMGB1 (GTX-101277, Genetex, 1:1,000), mouse anti-gasdermin-D (GSDMDC1 (A-7), Santa Cruz Biotechnology, 1:1,000; Supplementary Fig. 7), rabbit anti-gasdermin-D (1:2,000, G7422, Sigma, all other figures) and mouse anti-β-actin (A1987, Sigma, 1:1,000).

### Co-immunoprecipitation

Co-immunoprecipitation was done according to a modified protocol published previously^49^. For assessment of ASC/AIM2 interactions, 3 × 800,000 HEK293T cells were transfected with 2 μg per well AIM2-V5 and ASC-mCherry (ASC^FL^, ASC^K21A^, ASC^Y59A^ or ASC^E80R^) encoding plasmids at a ratio of 1:4 using linear polyethylenimine (PEI, Polysciences) at a ratio DNA:PEI of 1:4 (refs 65, 66). Twenty-four hours after transfection, the cells were washed twice with ice-cold 1 × PBS and lysed using HEK lysis buffer (20 mM HEPES pH 7.4, 10 mM KCl, 1 mM EDTA, 0.1 mM phenylmethylsulfonyl fluoride, 1 mM Na_3_VO_4_, 5 mM NaF, 0.5 % Nonidet P-40 and 1 × protease inhibitor cocktail (Roche)). Lysates were sonicated for 5 × 7 s before removing debris and non-lysed cells by centrifugation (10,000 *g*, 15 min, 4 °C). The samples were incubated with 1 μg mouse anti-V5 antibody (R960-25, Invitrogen) or a control antibody (mouse anti-GFP, 632381, Clontech) with agitation for 16 h at 4 °C. For assessment of ASC^CARD^/ASC^CARD^ interactions, 3 × 800 000 HEK293T cells were transfected with 1 μg per well ASC-mCherry (ASC^FL^, ASC^PYD^, ASC^D130R^ or ASC^D134R^), 1 μg per well ASC^CARD^-GFP and 0.2 μg AIM2-V5, to initiate ASC/ASC interactions using linear PEI. Forty hours after transfection, the cells were washed, lysed and sonicated as described above. The samples were then incubated with 1 μg mouse anti-mCherry antibody (ab125096, Abcam) or a control antibody (mouse anti-HA, MMS-101R-200, Covance). The samples were then incubated with agitation for 2 h at 4 °C with 25 μl Pierce Protein A Plus Agarose bead slurry (22810, Thermo Scientific). Beads were washed three times with lysis buffer (centrifugation 1 min 1,000 *g*, 4 °C), resuspended in 2 × SDS–PAGE sample buffer (30 μl) and boiled at 95 °C, before analysing the eluted proteins by western blotting as described above.

### CRISPR-Cas9-mediated *Caspase1* and *Gasdermin-D* knockout

Two guide RNAs^52^ targeting exon 4 of *Casp1* (5′-gagggcaagacgtgtacgag-3′ and 5′-cgagtggttgtattcattat-3′) and one guide RNA targeting exon 2 of *Gsdmd* (5′-ggtcaagaatgtgatcaagg-3′) were cloned into lentiCRISPRv2 harbouring a puromycin resistance cassette (Addgene^53^). These constructs were transfected into HEK293T cells using PEI (as described above) together with the lentiviral packaging vector PsPax2 (Addgene) and the lentviral envelope vector VSV-G (Addgene). Sixteen hours after transfection, medium was exchanged with macrophage medium and incubated at 37 °C for 2 days to produce lentiviral particles. The lentiviral particles were used to transfect the immortalized macrophage cell lines (800,000 cells per well in 6-well plates) using polybrene (Merck) to favour virus attachment. Two days after viral transduction, the macrophages were expanded. Attached macrophages were then treated with 10 μg ml^−1^ puromycin (Gibco) for 6 days to select for successful lentiviral transduction. After puromycin selection, the cells were tested for successful knockout by western blotting.

### Expression and purification of ASC^PYD^

The ASC^PYD^ (residues 1–91) was cloned with a C-terminal His_6_ tag into the pET28a vector under the control of a T7 promoter. Protein expression was induced by isopropyl-β-D-thiogalactopyranosid addition in BL21(DE3) *Escherichia coli* at an OD_600_ of 0.8 for 4 h at 37 °C. Bacteria were harvested by centrifugation and resuspended in 50 mM phosphate buffer pH 7.5, 300 mM NaCl, with Complete protease inhibitor (Roche). Resuspended bacteria were incubated for 1 h at room temperature with DNase I, sonicated on ice and centrifuged at 20,000 *g* at 4 °C for 30 min. The pellet, including ASC^PYD^-containing inclusion bodies, was solubilized in 50 mM phosphate buffer pH 7.5, 300 mM NaCl, 6 M guanidinium hydrochloride and centrifuged at 20,000 *g* at 4 °C for 30 min. The supernatant was incubated for 2 h at room temperature with pre-equilibrated Ni-NTA affinity resin (Thermo Scientific) and then passed through a plastic body column for gravity flow purification. The column was washed with 20 column volumes of solubilization buffer containing 20 mM imidazole and eluted with 3 column volumes of solubilization buffer with 500 mM imidazole. The pH of the elution fraction was decreased to 3.8 and dialysed against 50 mM glycine buffer pH 3.8, 150 mM NaCl. The protein was further purified on a pre-equilibrated Superdex 75 gel filtration column (GE Healthcare). This gel-filtration step removed traces of pre-existing aggregates and yielded highly pure, monomeric soluble form of ASC^PYD^. Samples were either used immediately or stored after flash-freezing in small aliquots in liquid N_2_.

### Measurements of ASC^PYD^ filament formation kinetics *in vitro*

Immediately before the experiments, samples of monomeric soluble ASC^PYD^ were centrifuged at 20,000 *g* at 4 °C for 30 min and filtered with 0.1 μM filter (Millipore). The protein concentration was adjusted to 25 μM by dilution from a higher-concentrated stock solution. Filament formation was triggered by rapid dilution to neutral pH. Thereby, 70 μl of monomeric ASC^PYD^ was mixed with 0.45 μl of 2.75 M NaOH solution to a reach the pH of 7.5. The solution was mixed at room temperature by careful pipetting, to avoid introduction of air bubbles, and immediately transferred to a quartz cuvette with 1 cm path length. Between runs, cuvettes were carefully cleaned with 1 M Hellmanex solution (Sigma-Aldrich) to avoid cross-seeding effects between sequential measurements. Filament growth was monitored by dynamic light scattering with a Malvern Zetasizer Nano ZS series instrument. The laser focal spot was positioned in the middle of the cuvette and maintained fixed for all the measurements. To maximize the intensity of the scattered light, the minimal attenuation level was used. Data were acquired in 60 s intervals by averaging three runs of 20 s, until a total time of 350 min. Afterwards, the protein solution was blotted on EM grids, negatively stained and imaged with transmission electron microscopy to visualize filament formation.

### Data analysis

Filament growth was modelled assuming pseudo-first-order kinetics where the filament propagation step occurs by the addition of monomers to the initial growth centre. The time-dependent growth signal *I* was fitted independently for each measurement by a single exponential function,


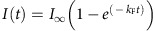


where *I*_∞_ corresponds to the signal at time *t* and at infinitive time, respectively, and *k*_F_ is the first-order rate constant. Fits were done with nonlinear least-square minimization.

### Data availability

The data that support the findings of this study are available from the corresponding author upon request.

## Additional information

**How to cite this article:** Dick, M. S. *et al*. ASC filament formation serves as a signal amplification mechanism for inflammasomes. *Nat. Commun.* 7:11929 doi: 10.1038/ncomms11929 (2016).

## Supplementary Material

Supplementary Information Supplementary Figures 1-9, Supplementary Table 1 and Supplementary References.
