# Corrigendum: ASC filament formation serves as a signal amplification mechanism for inflammasomes

**DOI:** 10.1038/ncomms15030

**Published:** 2017-03-17

**Authors:** Mathias S. Dick, Lorenzo Sborgi, Sebastian Rühl, Sebastian Hiller, Petr Broz

Nature Communications 7: Article number: 11929; DOI: 10.1038/ncomms11929 (2016); Published 06 22 2016; Updated 03 17 2017

In this Article, residues D128 and D132 of ASC are consistently referred to incorrectly as D130 and D134, respectively. These errors appear in the Results, Methods, Fig. 2, Fig. 3 and Supplementary Fig. 1.

