# Supplementary material for: ASC filament formation serves as a signal amplification mechanism for inflammasomes
Source: Nat Commun. 2016 Jun 22;7:11929. doi: 10.1038/ncomms11929 (PMC4917984; doi:10.1038/ncomms11929)
Supplement: Supplementary Information — Supplementary Figures 1-9, Supplementary Table 1 and Supplementary References. [file ncomms11929-s1.pdf]

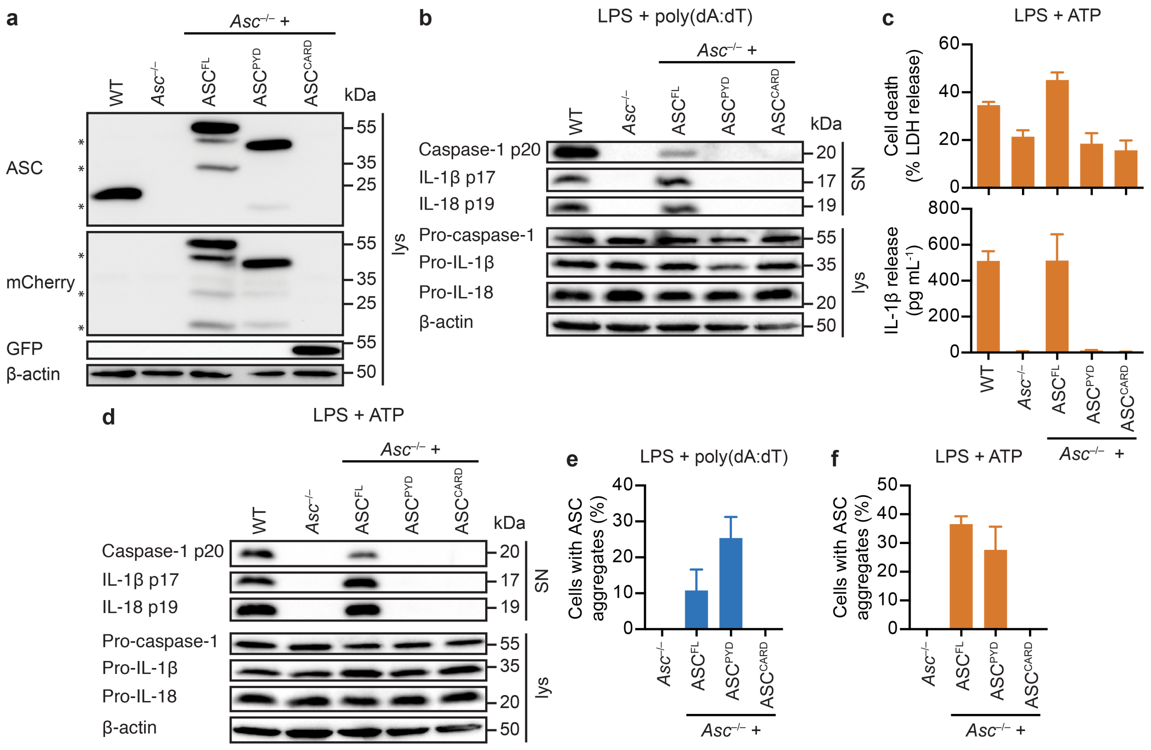

Supplementary Figure 1 (Related to Fig. 1)

(a) Expression of the ASC constructs in immortalized *Asc*$^{-/-}$ BMDM cell lysates (lys) as assessed by immunoblotting against eGFP, mCherry, ASC and β-actin. * denotes degradation bands resulting from a degraded mCherry, but with intact ASC. (b) Immunoblot analysis for cleaved caspase-1 p20, IL-1β p17 and IL-18 p19 in cell supernatants (SN) and pro-caspase-1, pro-IL-1β, pro-IL-18 and β-actin in lysates (lys) of LPS-primed immortalized wildtype (WT), *Asc*$^{-/-}$ or *Asc*$^{-/-}$ BMDMs expressing wildtype ASC (ASC^{FL}), ASC^{PYD} or ASC^{CARD} 3 h after poly(dA:dT) transfection (1 µg ml^{-1}). (c) Release of LDH and IL-1β from LPS-primed immortalized wildtype, *Asc*$^{-/-}$ or *Asc*$^{-/-}$ BMDMs expressing ASC^{FL}, ASC^{PYD} or ASC^{CARD} 1 h after ATP treatment (5 mM). (d) Immunoblots analysis for cleaved caspase-1 p20, IL-1β p17 and IL-18 p19 in cell supernatants (SN) and pro-caspase-1, pro-IL-1β, pro-IL-18 and β-actin in lysates (lys) of LPS-primed immortalized wildtype, *Asc*$^{-/-}$ or *Asc*$^{-/-}$ BMDMs expressing ASC^{FL}, ASC^{PYD} or ASC^{CARD} 1 h after 5 mM ATP treatment. (e-f) Quantification of ASC aggregates (specks and filaments) in LPS-primed immortalized *Asc*$^{-/-}$ BMDMs

expressing ASC^{FL}, ASC^{PYD} or ASC^{CARD} 3 h after poly(dA:dT) transfection (1 μg ml^{-1}) (e) or 1 h after ATP treatment (5 mM) (f). Graphs show the mean and s.d. of quadruplicate wells or ten random fields of view. Results shown are representative of at least three (a, c, e-f) or two independent experiments (b, d). See also Supplementary Figure 9.

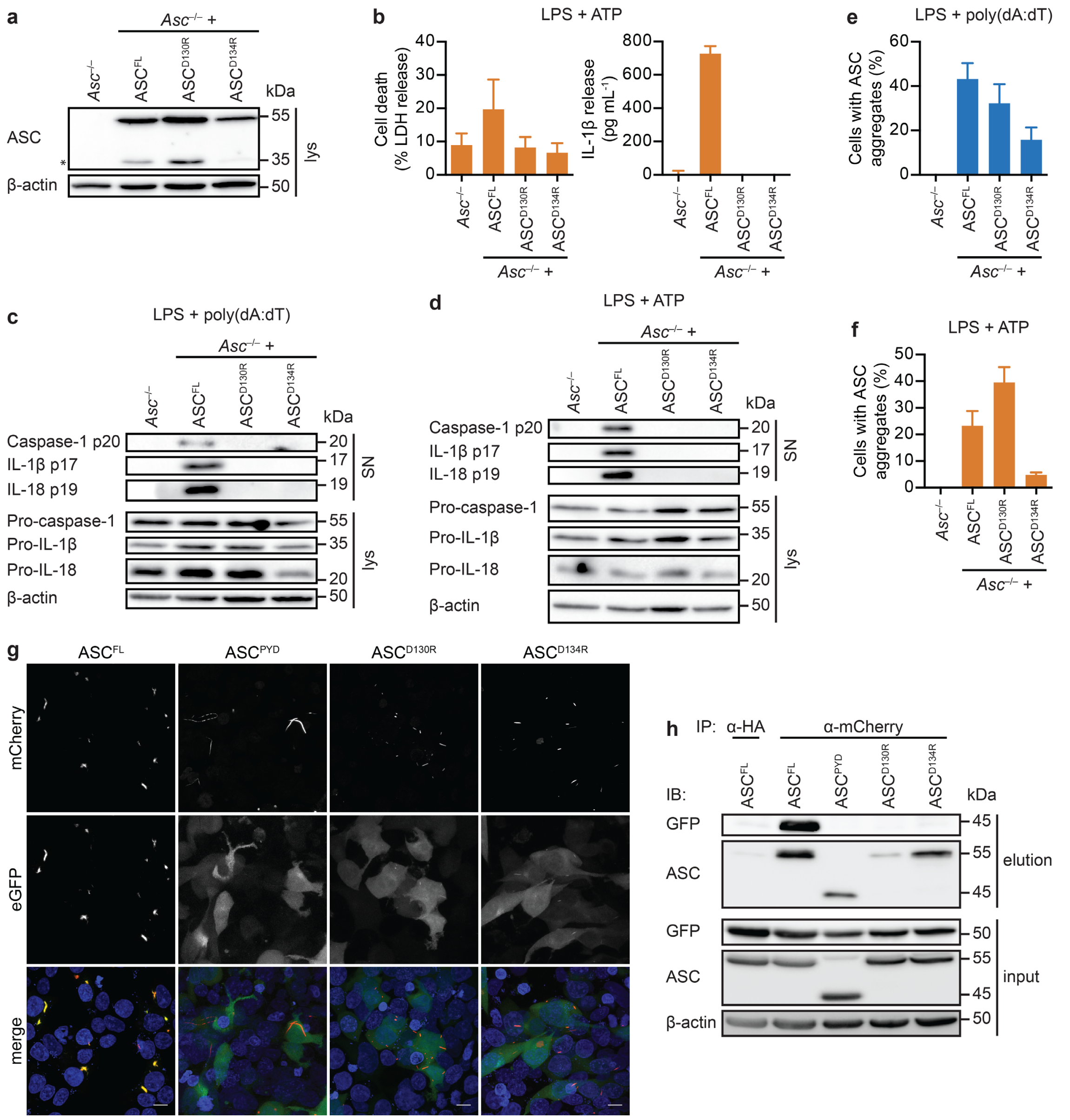

Supplementary Figure 2 (Related to Fig. 2)

(a) Equal expression of ASC^{CARD} mutants (ASC^{D130R} and ASC^{D134R}) in immortalized *Asc*$^{-/-}$ BMDM cell lysates (lys) as assessed by immunoblotting against ASC and β-actin. * denotes a degradation band. (b) Release of LDH and IL-1β from LPS-primed immortalized *Asc*$^{-/-}$ BMDMs and *Asc*$^{-/-}$ BMDMs expressing ASC^{FL}, ASC^{D130R} or ASC^{D134R} 1 h after ATP treatment (5 mM). (c-d) Immunoblots analysis for cleaved caspase-1 p20, IL-1β p17 and IL-18 p19 in cell supernatants (SN) and pro-caspase-1, pro-IL-1β, pro-IL-18 and β-actin in lysates

(lys) of LPS-primed immortalized $Asc^{-/-}$ BMDMs and $Asc^{-/-}$ BMDMs expressing ASC^{FL}, ASC^{D130R} or ASC^{D134R} 3 h after poly(dA:dT) transfection (1 μg ml^{-1}) (c) or 1 h after 5 mM ATP treatment (5 mM) (d). (e-f) Quantification of ASC aggregates (specks and filaments) from LPS-primed immortalized $Asc^{-/-}$ BMDMs and $Asc^{-/-}$ BMDMs expressing ASC^{FL}, ASC^{D130R} or ASC^{D134R} 3 h after poly(dA:dT) transfection (e) or 1 h after ATP treatment (5 mM) (f). (g) Co-localization of mCherry-tagged ASC^{FL}, ASC^{PYD}, ASC^{D130R} and ASC^{D134R} (red) with GFP-tagged ASC^{CARD} (green) overexpressed in HEK293T cells together with AIM2-V5 (to induce inflammasome assembly, not shown). DNA was stained with Hoechst (blue). Scale bars 10 μm. (h) Immunoblot analysis of the interaction of ASC^{CARD} with ASC^{FL}, ASC^{PYD}, ASC^{D130R} or ASC^{D134R}. ASC-mCherry was immunoprecipitated from lysates of HEK293T cells co-transfected with AIM2-V5, ASC^{CARD}-GFP and the indicated ASC-mCherry constructs. Co-immunoprecipitating proteins were identified using anti-GFP, anti-ASC and anti-β-actin antibodies. Graphs show the mean and s.d. of quadruplicate wells or ten random fields of view. Results shown are representative of at least two (a, c-d, g-h) or three (b, e-f) independent experiments. See also Supplementary Figure 9.

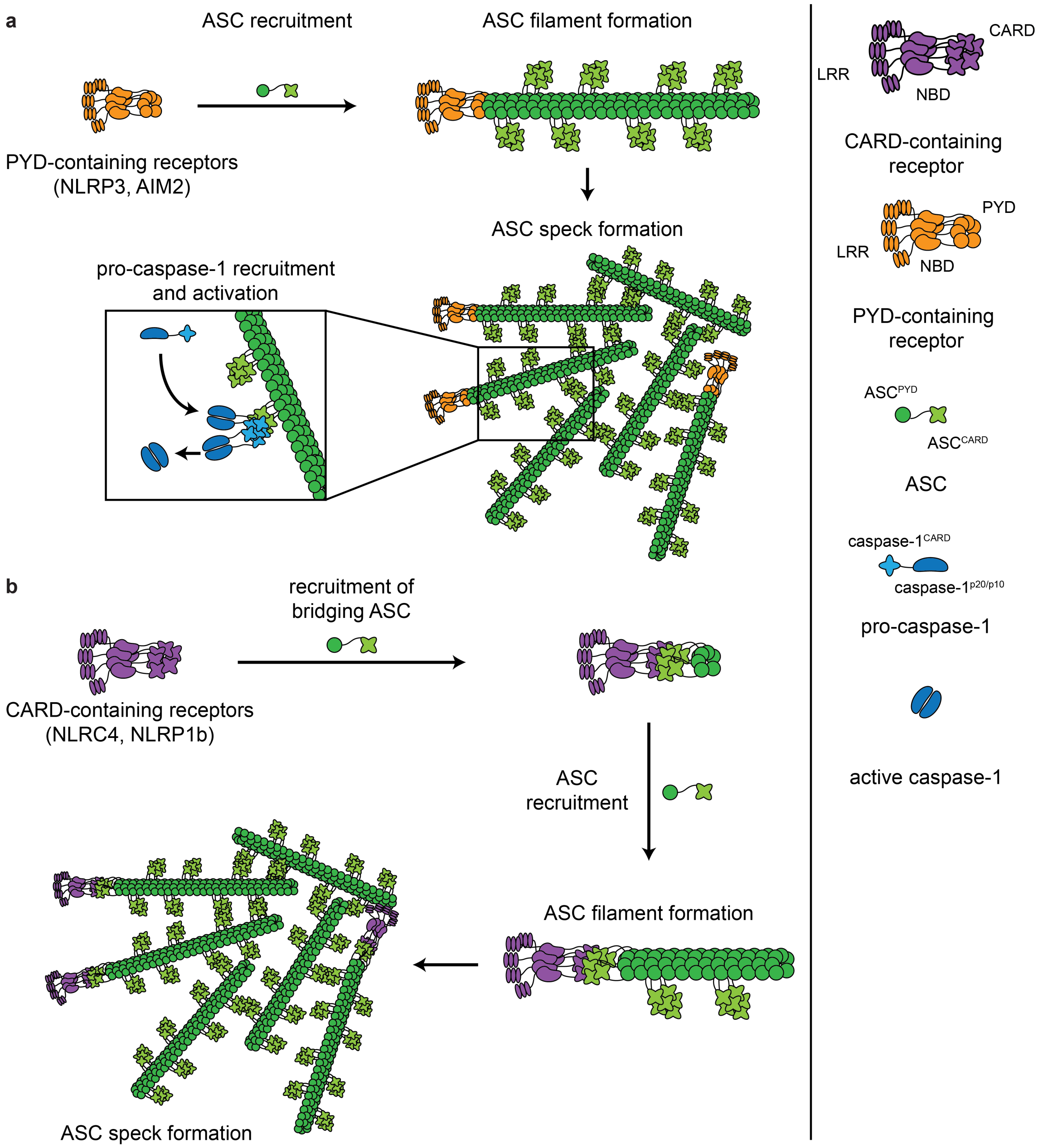

Supplementary Figure 3. Model describing ASC filament initiation and ASC speck assembly. (Related to Fig. 2 and Fig. 3)

(a) Activation of PYD-containing inflammasome receptors leads to the recruitment of ASC by homotypic receptor^{PYD}–ASC^{PYD} interaction and nucleation of ASC^{PYD} filaments. ASC^{CARDs} are exposed on the surface of ASC^{PYD} filaments. CARD–CARD interactions between ASC^{CARDs} of different filaments connect the different ASC^{PYD} filaments and condense them into the dense ASC speck

structure. At the same time surface exposed ASC^{CARD} serves as the recruitment and activation point for pro-caspase-1. (b) CARD–containing receptors recruit ASC bridging molecules by homotypic $receptor^{CARD}$–ASC^{CARD} interactions. The PYDs of the ASC bridging molecules act as a nucleation point for ASC^{PYD} filaments, a function that is otherwise executed by the $receptor^{PYD}$, as described in (a). The different ASC^{PYD} filaments are then condensed into the ASC speck by the ASC^{CARD}.

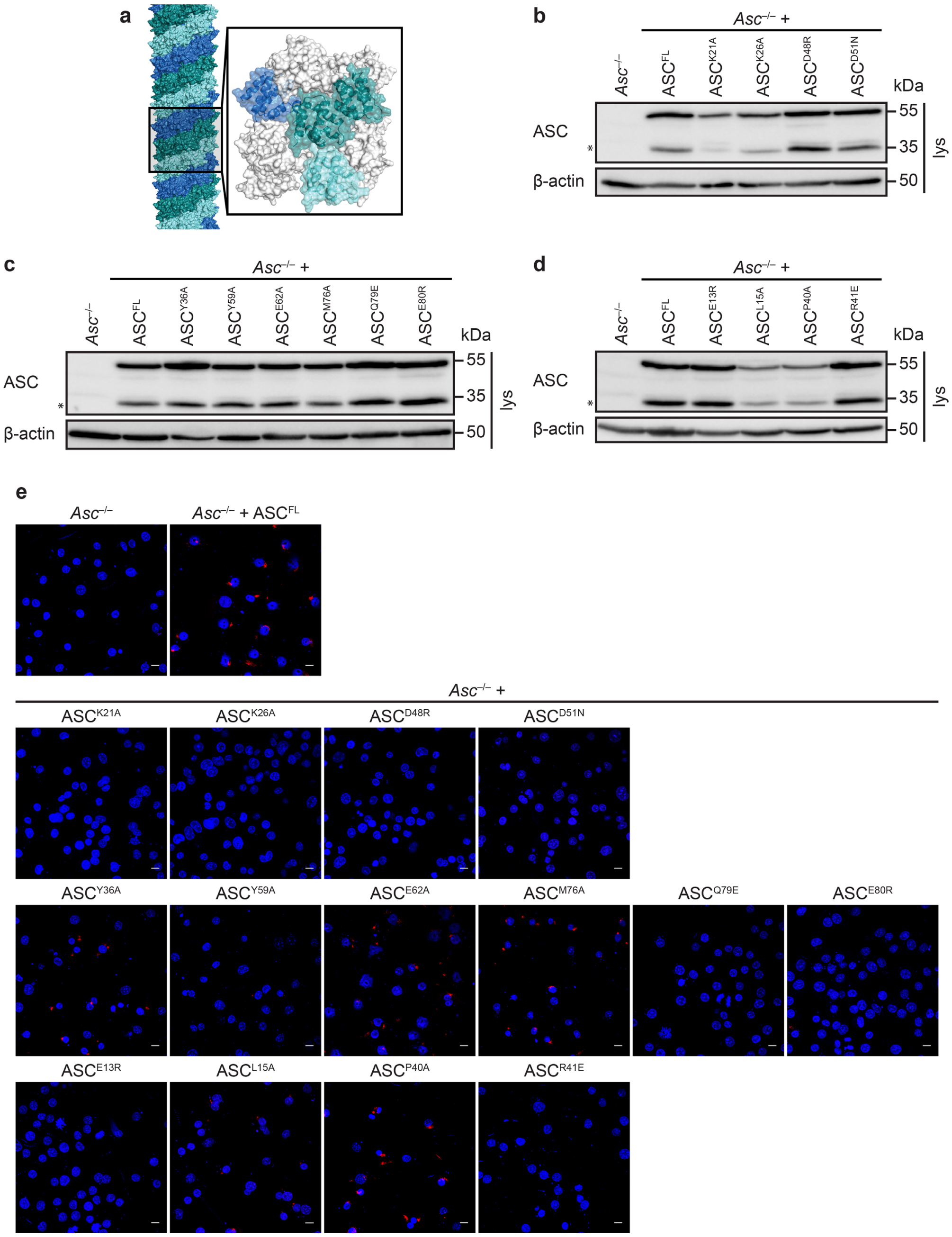

Supplementary Figure 4 (Related to Fig. 4)

a) Structure of the mouse ASC^{PYD} filament [PDB 2N1F[1]]. The three helical layers are colored blue, teal and light blue. A zoom up view shows the surface representation of ASC^{PYD} monomers as part of the filament. (b-d) Equal expression of wildtype ASC (ASC^{FL}) and ASC interaction interface I (b), interface II (c) and interface III (d) mutants in immortalized *Asc*$^{-/-}$ BMDM cell lysates (lys) as assessed by immunoblotting using anti-ASC and anti-β-actin antibodies. *

denotes a degradation band. (e) Representative images from LPS-primed immortalized *Asc*$^{-/-}$ BMDMs expressing mCherry-tagged ASC^{FL} or the indicated ASC interaction interface mutants after 3 h of poly(dA:dT) transfection (1 μg ml^{-1}). DNA was stained with Hoechst (blue), ASC (red). Scale bars 10 μM. Results shown are representative of two (b-d) or three (e) independent experiments. See also Supplementary Figure 9.

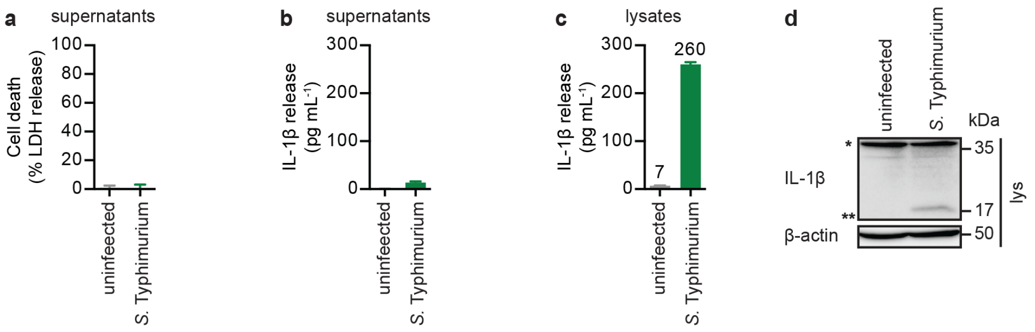

Supplementary Figure 5. **The IL-1β ELISA kit is more specific for the cleaved IL-1β p17.**

(a-b) Release of LDH (a) and IL-1β from LPS-primed immortalized *Gsdmd*$^{-/-}$ BMDMs left uninfected or infected with log phase *S*. Typhimurium SL1344 (MOI 10 for 1 h). (c) Release of IL-1β as detected by the ELISA kit used for this study (Mouse IL-1 beta ELISA Ready-SET-Go!® from eBiosciences, CatNo. 88-7013) from the cells in (a) and lysed by freezing and thawing in water. Numbers above bars indicate the average values. (d) Immunoblot analysis for pro-IL-1β (*) and bioactive IL-1β p17 (**) in lysates of the cells as in (c). Graphs show the mean and s.d. of quadruplicate wells and results shown are representative of at least three independent experiments. See also Supplementary Figure 9.

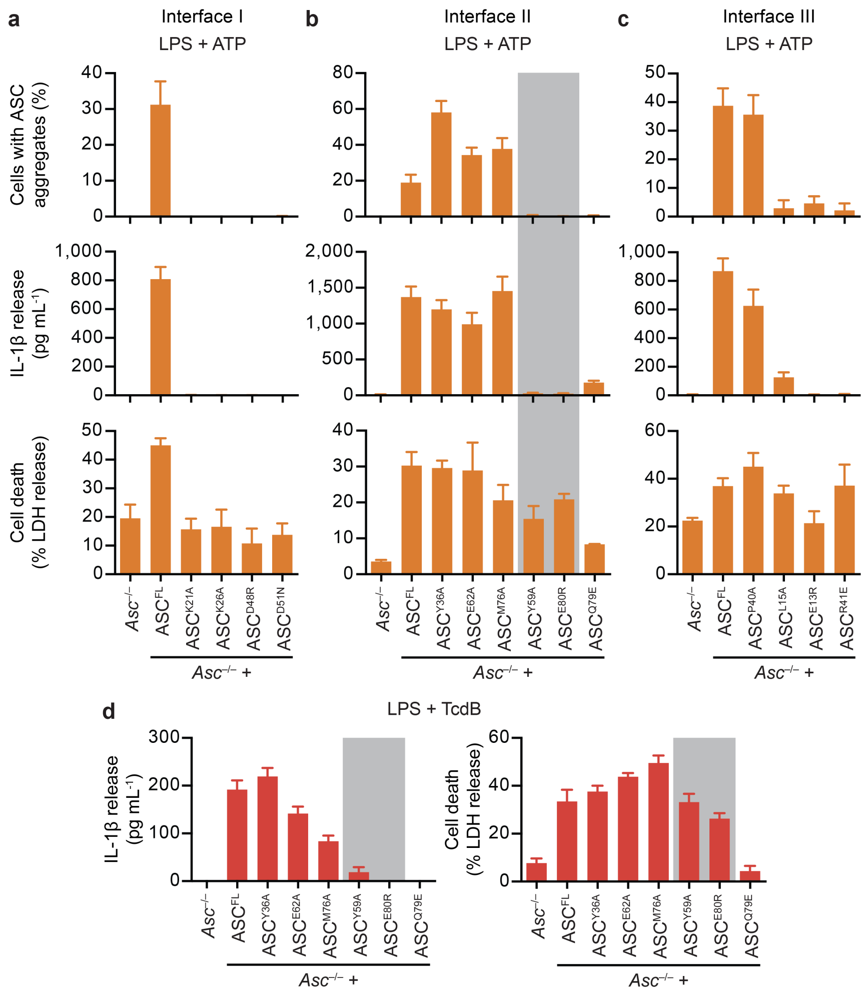

Supplementary Figure 6. Cell death is uncoupled from IL-1β release in NLRP3 and PYRIN inflammasomes (Related to Fig. 4)

(a-c) Quantification of ASC aggregates or the release of LDH and IL-1β from LPS-primed immortalized *Asc*$^{-/-}$ BMDMs and *Asc*$^{-/-}$ BMDMs expressing ASC^{FL} or the indicated ASC mutants 1 h after ATP treatment (5 mM). (d) Release of LDH and IL-1β from LPS-primed immortalized *Asc*$^{-/-}$ BMDMs and *Asc*$^{-/-}$ BMDMs expressing ASC^{FL} or the indicated ASC interface type II mutants 2.5 h after treatment with *Clostridium difficile* toxin B (TcdB, 1 μg ml^{-1}). ASC^{Y59A} and ASC^{E80R} are highlighted in grey. Graphs show mean and s.d. from quadruplicate wells or ten random fields of view. Data are representative of at least three independent experiments.

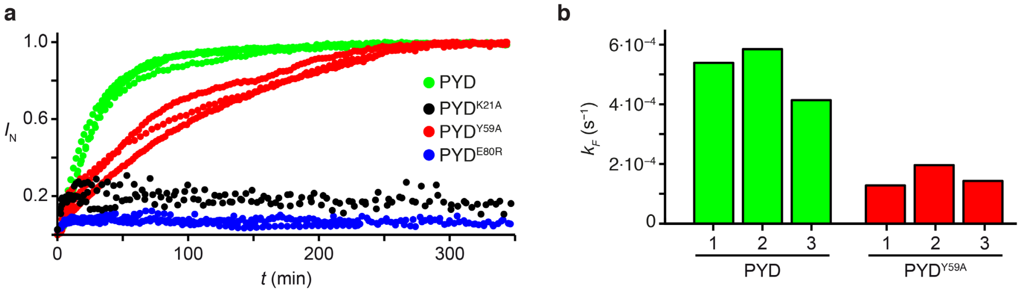

Supplementary Figure 7. Measurement of filament formation kinetics *in vitro* (Related to Fig. 5)

(a) Filament formation of wild-type ASC^{PYD} and its single amino-acid variants Y59A, K21A and E80R *in vitro* monitored by dynamic light scattering. Normalized growth signals (I_N) are reported as a function of time for three independent replicates of each variant (dotted lines). Three independent replicates are shown for each protein variant. Figure 5b displays one of these replicates. (b) Kinetic rate constants k_F of filament formation obtained from fitting the data in A with single exponential functions. Figure 5c displays the average and standard deviation of these data.

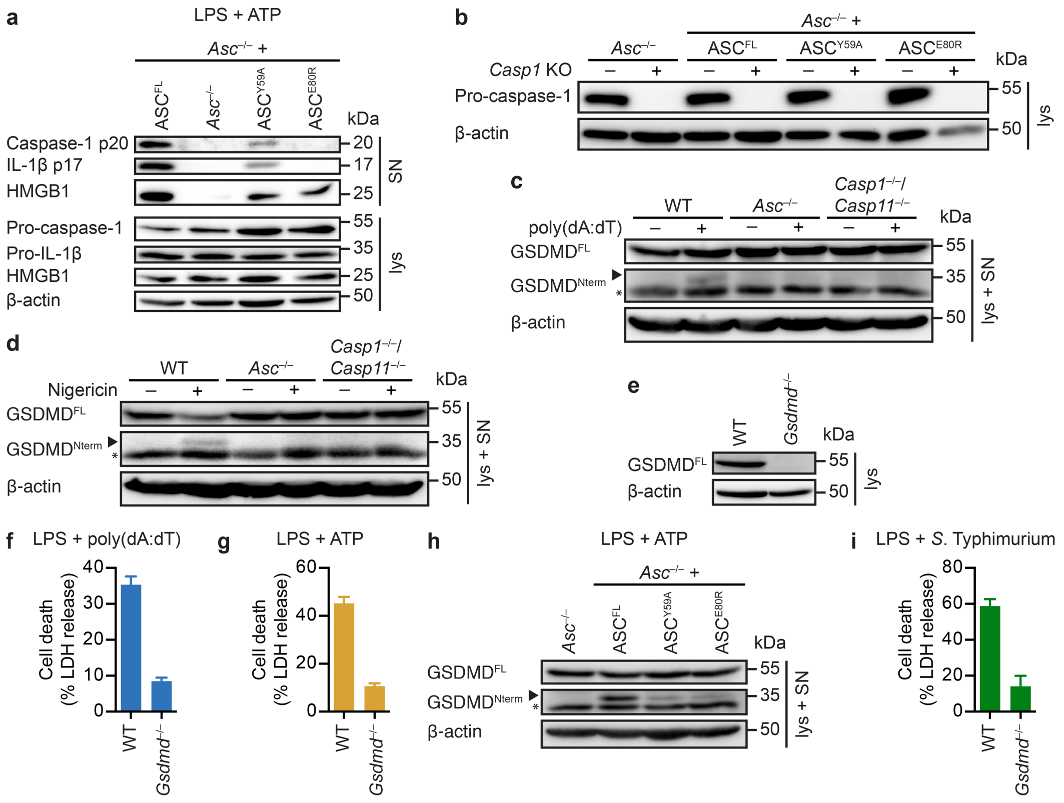

Supplementary Figure 8 (Related to Fig. 6)

(a) Immunoblot analysis for cleaved caspase-1 p20, IL-1β p17, and HMGB-1 in cell supernatants (SN) and pro-caspase-1, pro-IL-1β, and HMGB-1 in cell lysates (lys) of LPS-primed immortalized *Asc*$^{-/-}$ BMDMs or *Asc*$^{-/-}$ BMDMs expressing ASC^{FL}, ASC^{Y59A} or ASC^{E80R} after ATP treatment (5 mM, 1 h). (b) Confirmation of CRISPR-Cas9 mediated *Casp1* knock-out in immortalized *Asc*$^{-/-}$ BMDMs expressing ASC^{FL}, ASC^{Y59A} or ASC^{E80R} by immunoblotting. (c-d) Immunoblot analysis for processing of GSDMD into the active N-terminal fragment in combined lysates (lys) and supernatants (SN) of LPS-primed primary C57BL/6 WT (WT), *Casp1*$^{-/-}$/*Casp11*$^{-/-}$ *or Asc*$^{-/-}$ BMDMs transfected with 1 μg ml^{-1} poly(dA:dT) for 3 h (c), treated with 5 mM ATP for 1 h (d) or left untreated. (e) Confirmation of CRISPR-Cas9 mediated *Gsdmd* knock-out in immortalized WT BMDMs. (f-g) LDH release from immortalized WT or *Gsdmd*$^{-/-}$ BMDMs primed with LPS and then transfected with poly(dA:dT) (1 μg ml^{-1}, 3 h [f]) or treated with ATP (5 mM, 1 h [g]).

(h) Immunoblot analysis for processing of GSDMD into the active N-terminal fragment in combined lysates and supernatants (lys + SN) of LPS-primed immortalized *Asc*$^{-/-}$ BMDMs expressing ASC^{FL}, ASC^{Y59A} or ASC^{E80R} 1 h after ATP treatment (5 mM). (i) LDH release from immortalized WT or *Gsdmd*$^{-/-}$ BMDMs, primed with LPS and then infected with *S*. Typhimurium (MOI = 10, for 1 h). Arrowheads indicate the GSDMD^{Nterm} p30, * indicate a cross-reacting band. β-actin immunoblots serve as loading controls. Graphs show mean and s.d. from quadruplicate wells. Results shown are representative of two (a-b) or three (c-i) independent experiments. See also Supplementary Figure 9.

Figure 5a

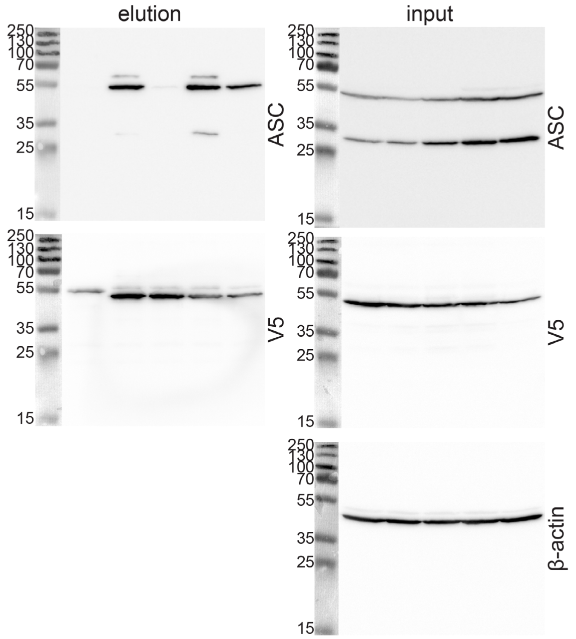

Figure 6a

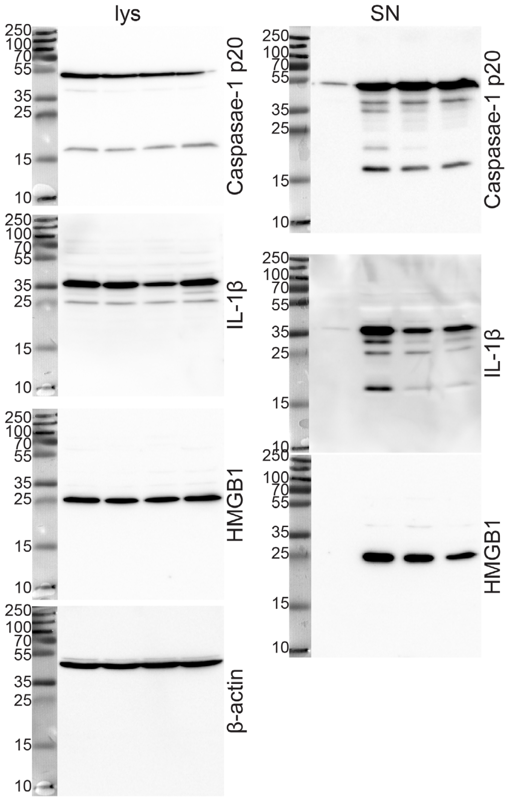

Fig. 6c

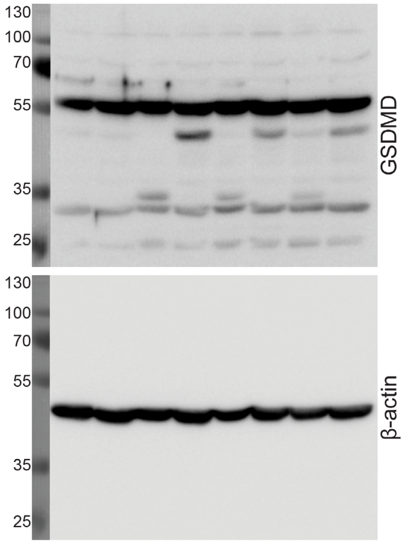

Fig. 6e

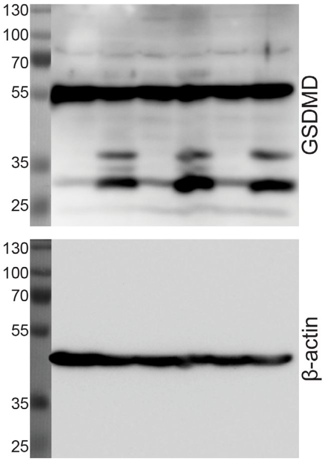

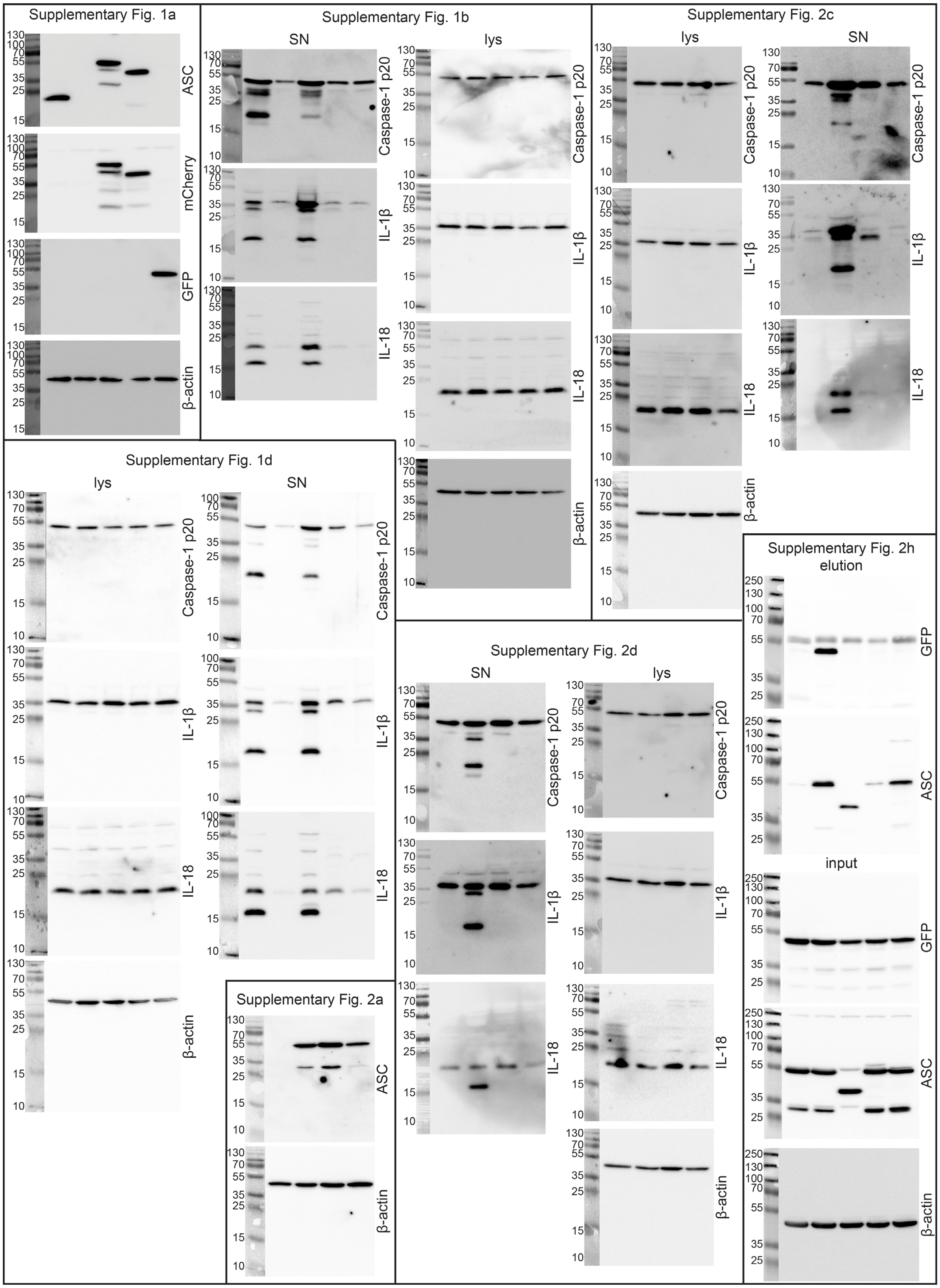
Supplementary Fig. 1a
ASC
mCherry
GFP
β-actin
Supplementary Fig. 1b
SN
lys
Caspase-1 p20
IL-1β
IL-18
β-actin
Supplementary Fig. 2c
lys
SN
Caspase-1 p20
IL-1β
IL-18
β-actin
Supplementary Fig. 1d
lys
SN
Caspase-1 p20
IL-1β
IL-18
β-actin
Supplementary Fig. 2d
SN
lys
Caspase-1 p20
IL-1β
IL-18
β-actin
Supplementary Fig. 2h
elution
GFP
ASC
input
GFP
ASC
β-actin
Supplementary Fig. 2a
ASC
β-actin

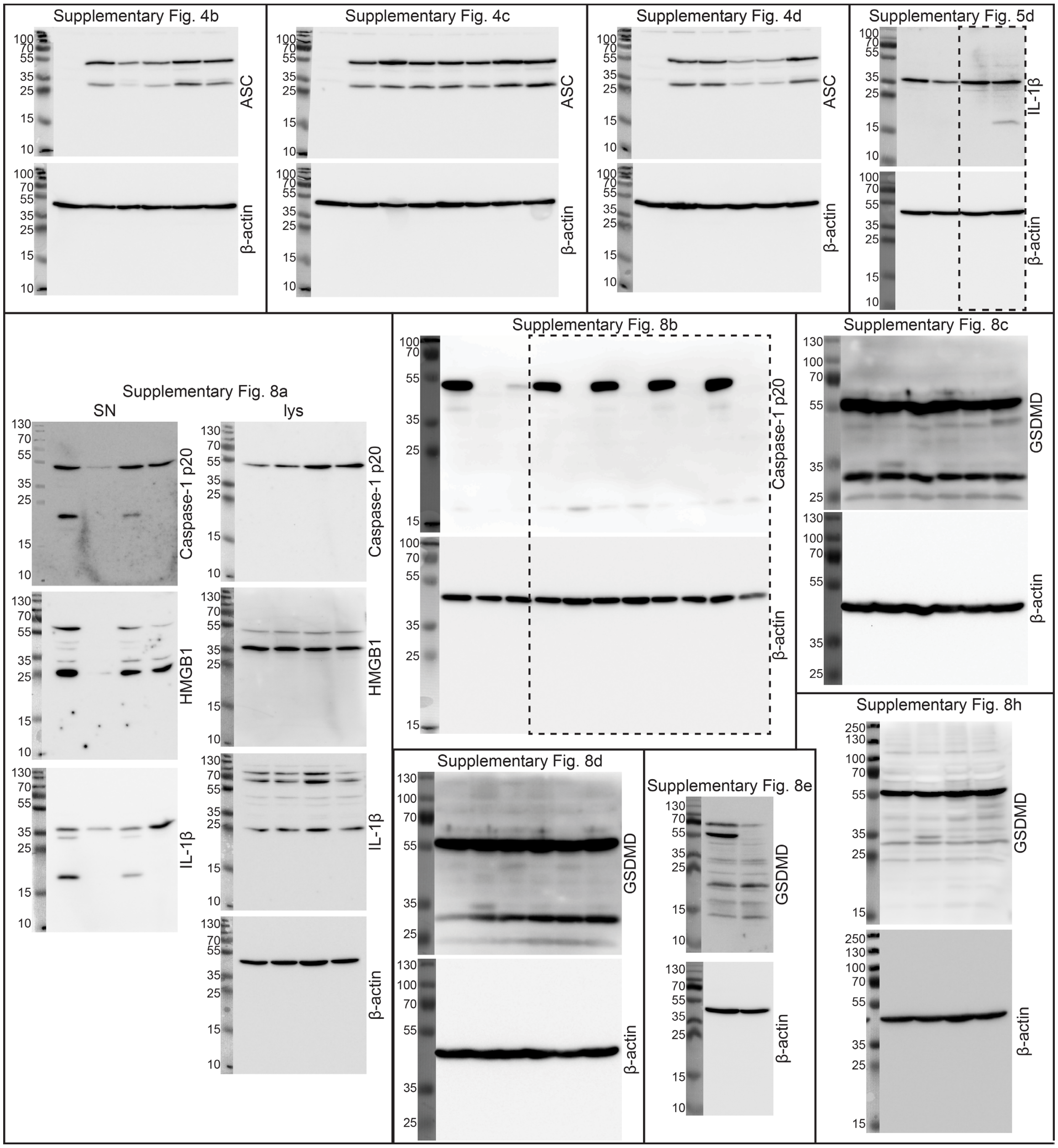

Supplementary Figure 9

Full images of blots shown in the main text and supplementary figures.

SUPPLEMENTARY TABLE

Supplementary Table 1. Oligonucleotides used for plasmid construction

Name	No.	Sequence	Restriction Site	Description
**ASC_D48R_fw**	O94	GCCCTGCTGCAGATGcgcGCCATAgatCTCACTGACAAAC		site directed mutagenesis, ASC^{D48R}
**ASC_D48R_rv**	O95	GTTTGTCAGTGAGatcTATGGCgcgCATCTGCAGCAGGGC		site directed mutagenesis, ASC^{D48R}
**ASC_D51N_fw**	O104	CTGCAGATGGACGCCATAaacCTCACTGACAAACTTGTCAGC		site directed mutagenesis, ASC^{D51N}
**ASC_D51N_rv**	O105	GCTGACAAGTTTGTCAGTGAGgttTATGGCGTCCATCTGCAG		site directed mutagenesis, ASC^{D51N}
**ASC_Y59A_fw**	O108	CACTGACAAACTTGTCAGCgccTATCTGGAGTCGTATGGCTTG		site directed mutagenesis, ASC^{Y59A}
**ASC_Y59A_rv**	O109	CAAGCCATACGACTCCAGATAggcGCTGACAAGTTTGTCAGTG		site directed mutagenesis, ASC^{Y59A}
**ASC_Y36A_fw**	O155	gcaactgcgagaaggcgccgggcgcatcccac		site directed mutagenesis, ASC^{Y36A}
**ASC_Y36A_rv**	O156	gtgggatgcgcccggcgccttctcgcagttgc		site directed mutagenesis, ASC^{Y36A}
**ASC_E62A_fw**	O159	gacaaacttgtcagctactatctgGCCtcgtatggcttggagctc		site directed mutagenesis, ASC^{E62A}
**ASC_E62A_rv**	O160	gagctccaagccatacgaGGCcagatagtagctgacaagtttgtc		site directed mutagenesis, ASC^{E62A}
**ASC_M76A_fw**	O161	caatgactgtgcttagagacGCCggcttacaggagctgg		site directed mutagenesis, ASC^{M76A}
**ASC_M76A_rv**	O162	ccagctcctgtaagccGGCgtctctaagcacagtcattg		site directed mutagenesis, ASC^{M76A}
**ASC_Q79E_fw**	O163	gtgcttagagacatgggcttaGAAgagctggctgagcag		site directed mutagenesis, ASC^{Q79E}
**ASC_Q79E_rv**	O164	ctgctcagccagctcTTCtaagcccatgtctctaagcac		site directed mutagenesis, ASC^{Q79E}
**ASC_E80R_fw**	O165	cttagagacatgggcttacagCGCctggctgagcagctg		site directed mutagenesis, ASC^{E80R}
**ASC_E80R_rv**	O166	cagctgctcagccagGCGctgtaagcccatgtctctaag		site directed mutagenesis, ASC^{E80R}
**ASC_E13R_fw_XhoI**	O243	GATCctcgagCCACCatggggcgggcacgagatgccatcctggacgctcttCGCaacttgtcag	XhoI	site directed mutagenesis, ASC^{E13R}
**ASC_R41E_fw**	O244	gggcgcatcccaGAAggggccctgc		site directed mutagenesis, ASC^{R41E}
**ASC_R41E_rv**	O245	gcagggccccTTCtgggatgcgccc		site directed mutagenesis, ASC^{R41E}
**ASC_L15A_fw_XhoI**	O246	GATCctcgagCCACCatggggcgggcacgagatgccatcctggacgctctt	XhoI	site directed mutagenesis, ASC^{L15A}

		gaaaacGCCtcaggggatg		
**ASC_P40A _fw**	O247	gctatgggcgcatcGCCcgcggggc cc		site directed mutagenesis, ASC^{P40A}
**ASC_P40A _rv**	O248	gggccccgcgGGCgatgcgcccata gc		site directed mutagenesis, ASC^{P40A}
**ASCfwXho I**	O7	GATCctcgagCCACCatggggcg ggcacgagatgc	XhoI	cloning of ASC into V48, forward
**ASC1-92rvEcoRI**	O4	GATCgaattcGctcttctttagtcgtttg cagct	EcoRI	cloning of ASC^{PYD} into V48, reverse
**ASC-CARD-GFPprimer A**	O21	GATCGCGGCCGCCACCATG GGAGCTGTGGCAGCTGCA G	NotI	cloning of ASC^{CARD}-GFPinto V48 by SOE PCR
**ASC-CARD-GFPprimer B**	O22	GATCCACCGGTCGCCACCA TGGTGAGCAAGGGCGAGG		cloning of ASC^{CARD}-GFPinto V48 by SOE PCR
**ASC-CARD-GFPprimer C**	O23	CCTCGCCCTTGCTCACCAT GGTGGCGACCGGTGGATC		cloning of ASC^{CARD}-GFPinto V48 by SOE PCR
**ASC-CARD-GFPprimer D**	O24	GATCGTTTAAACTTATCTAG ATCCGGTGGATCCC	PmeI	cloning of ASC^{CARD}-GFPinto V48 by SOE PCR
**RV_ascfl_ EcoRI**	O59	GATCgaattcggctctgctccaggtcc atca	EcoRI	cloning of ASC^{FL} into V48, reverse
**FWascD13 0R**	O13	CACAGAAGTGAGGGGAGTG CTG		site directed mutagenesis, ASC^{D130R}
**RVascD13 0R**	O14	CAGCACTCCCCTCACTTCT GTG		site directed mutagenesis, ASC^{D130R}
**FWascD13 4R**	O15	CGGAGTGCTGAGGGCTTTG CATG		site directed mutagenesis, ASC^{D134R}
**RVascD13 4R**	O16	CATGCAAAGCCCTCAGCAC TCCG		site directed mutagenesis, ASC^{D134R}

SUPPLEMENTARY REFERENCES

1. Sborgi, L. *et al.* Structure and assembly of the mouse ASC inflammasome by combined NMR spectroscopy and cryo-electron microscopy. *Proc. Natl. Acad. Sci.* (2015). doi:10.1073/pnas.1507579112
